# Elevating levels of the endocannabinoid 2-arachidonoylglycerol blunts opioid reward but not analgesia

**DOI:** 10.1126/sciadv.adq4779

**Published:** 2024-11-29

**Authors:** Arlene Martínez-Rivera, Robert N. Fetcho, Lizzie Birmingham, Jin Xu, Ruirong Yang, Careen Foord, Diego Scala-Chávez, Narmin Mekawy, Kristen Pleil, Virginia M. Pickel, Conor Liston, Carlos M. Castorena, Joshua Levitz, Ying-Xian Pan, Lisa A. Briand, Anjali M. Rajadhyaksha, Francis S. Lee

**Affiliations:** ^1^ Division of Pediatric Neurology, Department of Pediatrics, Weill Cornell Medicine, New York, NY 10065, USA.; ^2^ Feil Family Brain and Mind Research Institute, Weill Cornell Medicine, New York, NY 10065, USA.; ^3^ Center for Substance Abuse Research and Department of Neural Sciences, Lewis Katz School of Medicine at Temple University, Philadelphia, PA 19140, USA.; ^4^ Department of Psychiatry, Weill Cornell Medicine, New York, NY 10065, USA.; ^5^ Department of Psychology and Neuroscience Program, Temple University, Philadelphia, PA 19122, USA.; ^6^ Department of Anesthesiology, Rutgers New Jersey Medical School, Newark, NJ 07103, USA.; ^7^ Department of Pharmacology, Weill Cornell Medicine, New York, NY 10065, USA.; ^8^ Center for Hypothalamic Research, Department of Internal Medicine, University of Texas Southwestern Medical Center, Dallas, TX 75390, USA.; ^9^ Department of Biochemistry, Weill Cornell Medicine, New York, NY 10065, USA.

## Abstract

Converging findings have established that the endocannabinoid (eCB) system serves as a possible target for the development of new treatments as a complement to opioid-based treatments. Here, we show in male and female mice that enhancing levels of the eCB, 2-arachidonoylglycerol (2-AG), through pharmacological inhibition of its catabolic enzyme, monoacylglycerol lipase (MAGL), either systemically or in the ventral tegmental area (VTA) with JZL184, leads to a substantial attenuation of the rewarding effects of opioids in mice using conditioned place preference and self-administration paradigms, without altering their analgesic properties. These effects are driven by cannabinoid receptor 1 (CB1R) within the VTA, as VTA CB1R conditional knockout counteracts JZL184’s effects. Using fiber photometry with fluorescent sensors for calcium and dopamine (DA), we find that enhancing 2-AG levels diminishes opioid reward–related nucleus accumbens (NAc) activity and DA neurotransmission. Together, these findings reveal that 2-AG diminishes the rewarding properties of opioids and provides a potential adjunctive therapeutic strategy for opioid-related analgesic treatments.

## Teaser

Enhancing endocannabinoid 2-AG levels in the VTA blunts opioid reward and associated NAc activity and dopamine release.

## INTRODUCTION

Opioids such as morphine and oxycodone are mainstay analgesics for the management of moderate and severe acute pain. Unfortunately, they are also highly rewarding and can lead to drug dependence, making them a major contributor to the current opioid epidemic with opioid drug overdoses being a leading cause of death for Americans under the age of 50 years (*1*, *2*). Thus, the current situation has highlighted the urgent need to develop non-opioid analgesic alternatives. An additional strategy is to develop adjunctive treatments that can specifically attenuate the rewarding but not the analgesic properties of opioids. One possible avenue is engaging the brain’s endocannabinoid (eCB) system that consists of two major neuromodulatory ligands, 2-arachidonoylglycerol (2-AG) and *N*-arachidonoylethanolamine (anandamide; AEA) (*3*, *4*). These ligands act via the cannabinoid receptor 1 (CB1R) in the brain and regulate the dopaminergic system, including the mesolimbic ventral tegmental area (VTA) to nucleus accumbens (NAc) circuit that is at the core of opioid reward (*5*, *6*), with elevations of NAc dopamine (DA) associated with the rewarding properties of opioids (*7*–*13*).

**Fig. 1.**
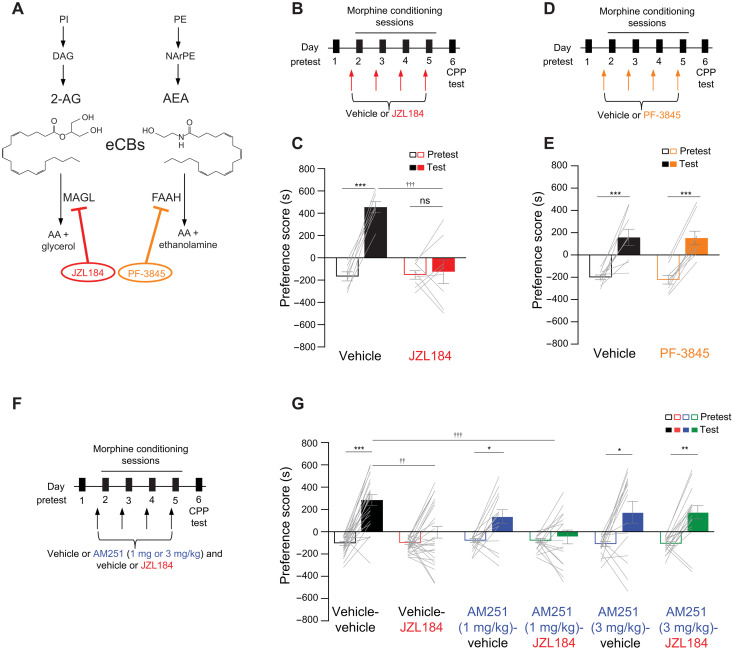
JZL184 attenuates morphine preference via CB1Rs. (**A**) eCB hydrolysis mechanism targeted by JZL184 and PF-385. (**B**) Timeline of morphine CPP and vehicle or JZL184 pretreatment. PI, phosphatidylinositol; DAG, diacylglycerol; PI, phosphatidylethanolamine; NAPE, N-arachidonoyl phosphatidylethanolamine; AA, arachidonic acid. (**C**) JZL184 abolished morphine CPP [two-way analysis of variance (ANOVA), significant interaction (treatment × day), *F*_1,26_ = 19.77, *P* = 0.0001; post hoc: vehicle: test versus pretest, ***P < 0.001; JZL184: test versus pretest, *P* > 0.9999; test: vehicle versus JZL184, †††*P* < 0.001; vehicle, *N* = 7; JZL184, *N* = 8]. (**D**) Timeline of morphine CPP and vehicle or PF-3845 pretreatment. (**E**) PF-3845 had no effect (two-way ANOVA, main effect of day, *F*_1,32_ = 48.49, *P* < 0.0001; post hoc: vehicle: test versus pretest, ***P < 0.001; PF-3845: test versus pretest, ***P = 0.001; vehicle, *N* = 9; PF-3845, *N* = 9). (**F**) Timeline of morphine CPP and AM251 and/or JZL184 pretreatment. (**G**) AM251 (3 mg/kg) before JZL184 counteracted JZL184 effects on morphine CPP {three-way ANOVA, significant interaction [AM251 (3 mg/kg): AM251 × days × JZL184, *F*_1,78_ = 5.159, *P* = 0.0259; post hoc: vehicle-vehicle: test versus pretest, ***P < 0.001; vehicle-JZL184: test versus pretest, *P* > 0.9999; AM251-vehicle: test versus pretest, **P = 0.0053; AM251-JZL184: test versus pretest, **P = 0.0025; test: vehicle-vehicle versus vehicle-JZL184, †††*P* < 0.001]; three-way ANOVA, significant interaction [AM251 (1 mg/kg): AM251 × days, *F*_1,78_ = 4.131, *P* = 0.0456, JZL184 × days, *F*_1,77_ = 16.58, ***P =0.0001; post hoc: vehicle-vehicle: test versus pretest, ***P < 0.001; vehicle-JZL184: test versus pretest, *P* > 0.9999; AM251-vehicle: test versus pretest, *P = 0.0450; AM251-JZL184: test versus pretest, *P* > 0.9999; test: vehicle-vehicle versus vehicle-JZL184, †††*P* < 0.001; test: vehicle-vehicle versus AM251-JZL184, †††*P* < 0.001]; vehicle-vehicle, *N* = 24; vehicle-JZL184, *N* = 26; AM251 [3 mg/kg]–vehicle, *N* = 15; AM251 [3 mg/kg]–JZL184, *N* = 17; AM251 [1 mg/kg]–vehicle, *N* = 15; AM251 [1 mg/kg]–JZL184, *N* = 16}. Error bars ± SEM. ns, not significant.

eCB levels are tightly regulated by catabolic enzymes including monoacylglycerol lipase (MAGL) and fatty acid amide hydrolase (FAAH) which respectively hydrolyze 2-AG and AEA (Fig. 1A) (*14*). Recent studies using inhibitors of MAGL and FAAH have revealed an inhibitory role of eCBs on opioid reward. Notably, a dual FAAH-MAGL inhibitor (SA-57), which enhances both 2-AG and AEA levels, reduces heroin self-administration in male mice (*15*). Similarly, a separate study using the MAGL inhibitor MJN110, which enhances brain 2-AG levels (*16*), attenuates morphine place preference in male rats (*17*). Presumably, these inhibitory effects are via activation of CB1Rs, although this remains to be tested. Pharmacological blockade of CB1Rs produces a comparable inhibitory outcome, resulting in the reduction of heroin self-administration (*18*), thus presenting a perplexing paradox.

The complexity of the eCB system is further emphasized from studies on the effect of AEA and 2-AG on motivated behaviors and the DA system. While increasing AEA levels decreases cue-induced reward seeking and NAc DA (*19*), 2-AG has the opposite effect. Enhancing 2-AG with MAGL inhibition (JZL184) enhanced intracranial self-stimulation and food reward seeking as well as NAc DA in a CB1R-dependent manner (*19*), thus presenting a confounding/puzzling paradox.

Considering the divergent results on the role of eCBs on motivated reward-related behaviors and opioid reward, our study aimed to investigate the specific impact of enhancing 2-AG and AEA on opioid reward. We additionally assessed the dependency on CB1Rs and examined the effects on neural activity and DA release in the NAc. We found that pharmacologically inhibiting MAGL led to a substantial attenuation of the rewarding effects of morphine and oxycodone in male and female mice. Conversely, pharmacologically enhancing the levels of AEA had no effect on morphine reward. In addition, inhibiting MAGL locally in the VTA was sufficient to blunt morphine reward and diminished reward associated NAc activity and DA transmission. In summary, we have identified a unique interaction between 2-AG and the opioid system. Specifically, 2-AG selectively counteracts the rewarding properties of opioids via disruption of NAc reward-related neural activity and DA transmission while preserving their analgesic effects.

## RESULTS

### Pharmacologically inhibiting MAGL and not FAAH attenuates morphine preference

To study the effect of enhancing eCB levels on the rewarding properties of opioids, we used the pharmacological MAGL inhibitor JZL184, which increases 2-AG levels by inhibiting its hydrolysis (*20*), or the FAAH inhibitor PF-3845, which increases AEA levels by inhibiting its hydrolysis (Fig. 1A) (*21*). We initially tested the rewarding properties of morphine, a widely used mu-opioid receptor (MOR) agonist with strong reinforcing properties both in humans (*22*) and animals (*23*), using the conditioned place preference (CPP) assay, a well-established rodent behavioral proxy for drug reward (*24*). After an initial CPP pretest on day 1, adult male mice were systemically administered JZL184 [10 mg/kg, intraperitoneal (ip); Fig. 1B] or PF-3845 (10 mg/kg, ip; Fig. 1D) 2 hours before each of the four morphine conditioning sessions (days 2 to 5). Twenty-four hours later, male mice were tested for morphine preference (CPP test; day 6) in the absence of both the inhibitor and morphine (Fig. 1, B and D). We found that enhancing 2-AG with JZL184 administration during the conditioning phase of CPP attenuated morphine preference (Fig. 1C). A similar effect was observed in female mice (fig. S1, A and B). In contrast, enhancing AEA with PF-3845 at 10 mg/kg, ip (Fig. 1E), and 20 mg/kg, ip (fig. S1, C and D), in male mice, had no effect on morphine CPP, suggesting that the decrease in morphine preference is unique to 2-AG over AEA. On the other hand, JZL184 (10 mg/kg, ip) in male mice had no effect on locomotor activity (fig. S1E), open arm entries in the elevated plus maze (fig. S1F), or immobility in the tail suspension test (fig. S1G) compared to vehicle-treated animals. In addition, JZL184 on its own had no effect on morphine preference (fig. S1, H and I) in male mice, and pretreating male mice with JZL184 before the morphine CPP expression test also had no effect either (fig. S1, J and K). The doses and pretreatment time points of JZL184 and PF-3845, both irreversible covalent inhibitors (*25*, *26*), were based on previously published work (*27*, *28*).

Next, as 2-AG acts primarily through CB1Rs (*29*, *30*), we tested whether the effect of JZL184 was via CB1Rs. Of note, since MAGL has additional substrates (*31*), we wanted to confirm that the effects of JZL184 were driven by 2-AG, the most abundant of all MAGL substrates in the brain (*32*, *33*) and the only one that orthosterically bind CB1Rs (*34*). Male mice were injected with the CB1R antagonist AM251 (1 mg/kg, ip or 3 mg/kg, ip; Fig. 1, F and G), before treatment with vehicle or JZL184 during the morphine conditioning phase of CPP (Fig. 1F). As expected, JZL184 without AM251 as a pretreatment blunted morphine preference (Fig. 1G; vehicle-vehicle versus vehicle-JZL184). Pretreatment with 3 mg/kg, ip AM251 [Fig. 1G; vehicle-JZL184 versus AM251 (3 mg/kg, ip)–JZL184] but not 1 mg/kg, ip AM251 [Fig. 1G; vehicle-JZL184 versus AM251 (1 mg/kg)–JZL184] reversed JZL184’s effect on attenuating morphine preference, demonstrating that JZL184 acts via CB1Rs. Of note, AM251 (1 and 3 mg/kg, ip) on its own lowered morphine preference; however, mice acquired a preference that was not significantly different from vehicle-treated mice (Fig. 1G; AM251-vehicle versus vehicle-vehicle). Together, the above findings indicate that JZL184 blunts morphine preference via CB1Rs.

### Pharmacologically inhibiting MAGL attenuates oxycodone preference and self-administration

**Fig. 2.**
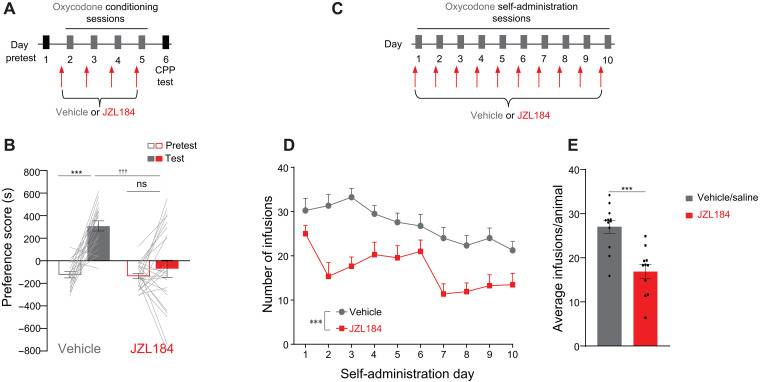
JZL184 attenuates oxycodone preference and self-administration. (**A**) Timeline of behavioral protocol for oxycodone CPP and systemic pretreatment of vehicle or JZL184. (**B**) Systemic JZL184 before each oxycodone conditioning session attenuated oxycodone CPP [two-way ANOVA, significant interaction (treatment × day), *F*_1,82_ = 13.31, *P* = 0.0005; post hoc: vehicle: test versus pretest ***P < 0.001; JZL184: test versus pretest, no significant; test: vehicle versus JZL184, †††*P* < 0.001; vehicle, *N* = 19; JZL184, *N* = 24]. (**C**) Timeline of behavioral protocol for oxycodone self-administration and systemic pretreatment of vehicle or JZL184. (**D** and **E**) Systemic JZL184 exposure before oxycodone self-administration sessions, attenuated the intake of oxycodone [(D) two-way RM ANOVA, main effect of JZL184 treatment, *F*_1,21_ = 21.43, ***P = 0.0001, main effect of days, *F*_2.962,62.20_ = 8.236, *P* = 0.0001, interaction *F*_9,189_ = 1.900, *P* = 0.0541; (E) average of total infusions per animal, *t* test, *t*_21_ = 4.63, ***P < 0.0001; vehicle/saline, *N* = 12; JZL184, *N* = 11]. Error bars ± SEM.

To test whether the effect of JZL184 is generalizable across opioids, we tested its effect on oxycodone CPP and self-administration. First, using CPP (Fig. 2A), we found that, similar to morphine, repeated dosing of JZL184 during oxycodone conditioning attenuated preference in adult male mice (Fig. 2B) without altering locomotor activity (fig. S2A). We next tested the effect of JZL184 on oxycodone self-administration (Fig. 2C). We chose to use oxycodone for the self-administration studies due to pharmacokinetic differences between morphine and oxycodone in mice. The shorter peak time and half-life of oxycodone seem to lead to higher consumption and less intoxication in the intravenous self-administration model. Male mice were first food-trained on the operant chamber for 2 days (fig. S2B). Then, mice were randomly separated into two groups, one pretreated with JZL184 (10 mg/kg, ip) and the other pretreated with vehicle or saline (no difference was observed between these two control groups; fig. S2, C and D); therefore, the groups were combined. Animals pretreated with JZL184 before each oxycodone-self administration session (0.25 mg/kg per infusion; Fig. 2C) self-administered less oxycodone when compared with the control group (Fig. 2, D and E). The percent active lever presses did not differ between groups (fig. S2, E and F). Similar results were obtained with female mice (fig. S2, G to L). These results demonstrate that JZL184 reduces the rewarding properties of oxycodone and that JZL184’s effects might be generalized across different opioids.

### Pharmacologically inhibiting MAGL does not alter opioid analgesia

**Fig. 3.**
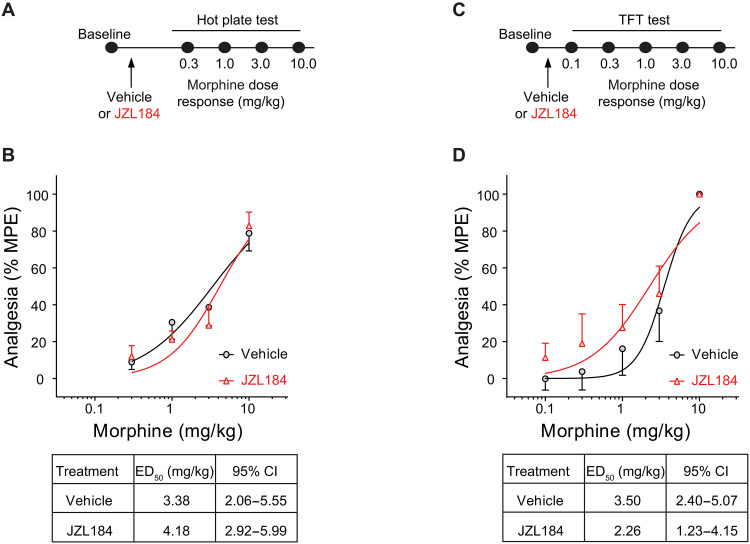
JZL184 has no effect on morphine analgesia. (**A** and **C**) Experimental timeline of acute systemic vehicle or JZL184 pretreatment before the morphine dose-response in the hot plate test (A) or the tail-flick test (C). (**B** and **D**) JZL184 pretreatment has no effect on morphine-induced analgesia during the hot plate test (B; two-way ANOVA, main effect of dose, *F*_3,68_ = 30.65, *P* < 0.001; vehicle, *N* = 9; JZL184, *N* = 10) or the tail-flick test (D; two-way ANOVA, main effect of dose, *F*_4,60_ = 22.17, *P* < 0.001; vehicle, *N* = 7; JZL184, *N* = 7). Error bars ± SEM.

Given the positive effects of JZL184 on attenuating opioid reward, we next queried whether JZL184 administration would alter the analgesic effects of morphine using the hot plate assay (*35*) and the radiant heat tail-flick test, behavioral assays commonly used to test pain response in animals (*36*). For the hot plate test, male mice were treated with a single dose of JZL184 (10 mg/kg, ip), and 2 hours later, a morphine dose-response (0.3, 1.0, 3.0, and 10 mg/kg, ip) test was performed (Fig. 3A). The half-maximal effective dose (ED_50_) value of vehicle-treated control animals was comparable to that in animals that received a single dose of JZL184 (Fig. 3B). Similarly, in the tail-flick test, a single dose of JZL184 had no effect on the ED_50_ value of morphine (Fig. 3, C and D) or oxycodone (fig. S3, A and B), suggesting that JZL184 had no effect on opioid-dependent analgesia.

We additionally examined whether repeated JZL184 administration, similar to the treatment regimen used in morphine CPP (fig. S3, C and D), would affect morphine analgesia. For this experiment, male mice were randomly separated into two groups and first subjected to a morphine dose-response to measure morphine analgesia (fig. S3D, middle/bottom; pre-vehicle and pre-JZL184 groups). Mice then received injections of vehicle or JZL184 (10 mg/kg, ip) once a day for 4 days. Twenty-four hours later, mice were retested for morphine analgesia (fig. S3D; post-vehicle and post-JZL184 groups). Similar to the above results, we did not observe significant differences between repeated JZL184 and vehicle-pretreated male mice. Similarly, to examine whether repeated JZL184 administration, equivalent to the regimen used in oxycodone self-administration (Fig. 2C), would affect oxycodone analgesia (fig. S3, E and F), we randomly assigned male mice into two different groups, and on day 1, mice were subjected to an oxycodone dose-response to measure analgesia (fig. S3F middle/bottom; pre-vehicle and pre-JZL184 groups). Then, mice received injections of vehicle or JZL184 (10 mg/kg, ip) once a day for 10 days. The following day, 24 hours after the last injection, mice were retested for oxycodone analgesia (day 12). There were no significant differences between pre- and post-JZL treatment (fig. S3F). Acute, 4-day or 10-day treatment with JZL184 on its own had no effect on the latency response at any time point tested (fig. S3, G and H). In addition, enhancing AEA with PF-3845 (10 mg/kg, ip; fig. S3, I and J) had no effect on morphine analgesia. Together, these data indicate that systemic elevation of 2-AG levels does not impair the ability of opioids to mediate analgesia in male mice. However, further pain assays are needed to confirm that JZL184 does not affect opioid analgesia.

### Inhibiting MAGL in the VTA blunts morphine preference but not analgesia

**Fig. 4.**
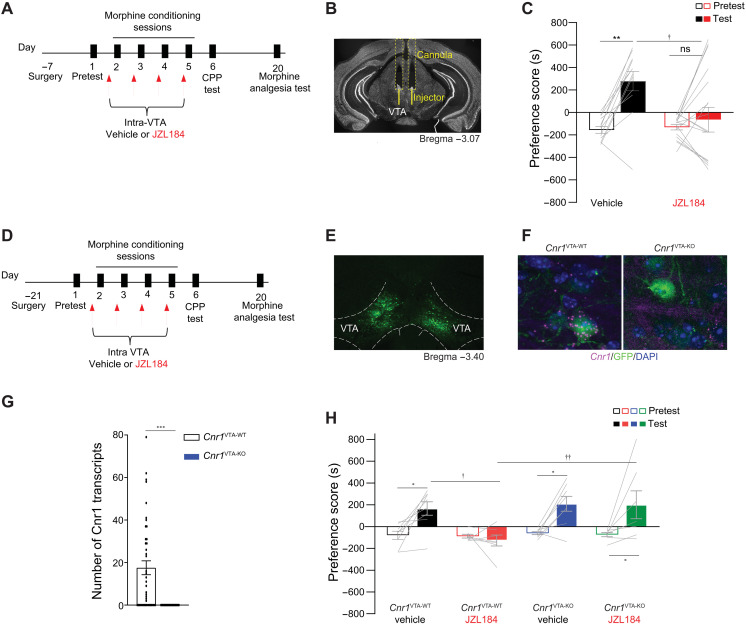
Intra-VTA JZL184 attenuates morphine preference, and VTA-CB1R knockout counteracts JZL184. (**A**) Experimental timeline for intra-VTA infusion of vehicle or JZL184 and morphine CPP. (**B**) Exemplar image of guide cannula placement in the VTA. (**C**) Intra-VTA of JZL184 attenuates morphine preference [two-way ANOVA, significant interaction (treatment × day), *F*_1,54_ = 5.144, *P* = 0.0274; post hoc: vehicle: test versus pretest, **P = 0.0051, JZL184: test versus pretest, *P* > 0.999; test: vehicle versus JZL184, †*P* = 0.018; vehicle, *N* = 12; JZL184, *N* = 17]. (**D**) Experimental timeline for surgery, morphine CPP, and tail-flick test. (**E**) Representative image of AAV2-Cre-GFP expression in the VTA. (**F**) Representative image of RNAscope showing *Cnr1* mRNA expression (magenta) and GFP-tagged cells (green) in the VTA of *Cnr1* ^VTA-WT^ (left) and *Cnr1* ^VTA-KO^ (right) mice (DAPI = blue). (**G**) Quantification of *Cnr1* puncta in *Cnr1*^VTA-WT^ and *Cnr1*^VTA-KO^ mice [*t* test, *t*_70_ = 4.508, ***P < 0.001; *Cnr1*^VTA-WT^, *N* = 3 (42 cells in total); *Cnr1*^VTA-KO^, *N* = 3 (25 cells in total)]. (**H**) Focal knockout of *Cnr1* in the VTA counteracts JZL184-induced blunting of morphine CPP [three-way ANOVA, significant interaction (days × genotype), *F*_1,26_ = 4.530, *P* < 0.043; post hoc: *Cnr1* ^VTA-WT^ vehicle: test versus pretest, *P = 0.044; *Cnr1* ^VTA-WT^ JZL184: test versus pretest, *P* > 0.999; *Cnr1* ^VTA-KO^ vehicle: test versus pretest, *P = 0.020; *Cnr1* ^VTA-KO^ JZL184: test versus pretest, *P = 0.031; test: *Cnr1* ^VTA-WT^ vehicle versus *Cnr1* ^VTA-WT^ JZL184, †*P* = 0.013; test: *Cnr1* ^VTA-WT^ JZL184 versus *Cnr1* ^VTA-KO^ JZL184, ††*P* = 0.005; *Cnr1* ^VTA-WT^ vehicle, *N* = 8; *Cnr1* ^VTA-WT^ JZL184, *N* = 7; *Cnr1* ^VTA-KO^ vehicle, *N* = 8; *Cnr1* ^VTA-KO^ JZL184, *N* = 7].

Having observed specific effects of inhibiting MAGL on opioid reward but not analgesia, we asked which brain region is involved in this effect. As the VTA is a central site for driving opioid reward (*5*), we tested whether the systemic effect of enhancing 2-AG was driven by its action locally in the VTA. JZL184 (5 μg/μl per side) was infused bilaterally into the VTA of male mice before each of the four morphine conditioning sessions (Fig. 4, A and B, and fig. S4A), and 24 hours later, mice were tested for morphine preference in the absence of both JZL184 and morphine. As was seen with systemic delivery (Fig. 1B), repeated application of intra-VTA JZL184 attenuated morphine preference (Fig. 4C). Furthermore, consistent with systemic JZL184 treatment, intra-VTA JZL184 infusion had no effect on morphine analgesia (fig. S4B), locomotor activity (fig. S4C), open arm entries in the elevated plus maze (fig. S4D), or immobility in the tail suspension test (fig. S4E), demonstrating that 2-AG is acting via effects on the VTA to specifically blunt opioid reward.

### Knockout of VTA CB1R blunts JZL184’s effects on morphine CPP

Next, to test whether CB1Rs within the VTA are responsible for JZL184’s effects on morphine CPP, we generated focal knockout of CB1Rs in the VTA using CB1R gene, *Cnr1* floxed mice. The VTA of *Cnr1* wild-type (*Cnr1*^WT^) or *Cnr1* floxed mice (*Cnr1*^fl/fl^) were bilaterally microinjected with AAV2-Cre-GFP to generate *Cnr1*^VTA-WT^ and *Cnr1*^VTA-KO^ mice (Fig. 4, D to F, and fig. S5A). To validate the efficiency of *Cnr1* knockout in virus-infected cells (Fig. 4E), we performed dual green fluorescent protein (GFP) immunofluorescence and *Cnr1* RNAscope in situ hybridization (Fig. 4F). Quantification of *Cnr1* puncta in *Cnr1*^VTA-WT^ and *Cnr1*^VTA-KO^ mice validated the viral strategy and confirmed the deletion of *Cnr1* mRNA in GFP-positive cells (Fig. 4G, RNAscope image quantification). *Cnr1*^VTA-WT^ and *Cnr1*^VTA-KO^ mice were subjected to morphine CPP receiving systemic JZL184 pretreatment before each morphine conditioning session (Fig. 4D). As expected, control *Cnr1*^VTA-WT^ animals with intact CB1Rs pretreated with vehicle acquired a preference for the morphine chamber, while JZL184-pretreated mice exhibited a blunted response (Fig. 4H). The blunted response was reversed in *Cnr1*^VTA-KO^ mice treated with JZL184 with these animals acquiring a preference for the morphine-paired chamber, demonstrating that VTA CB1Rs are necessary for JZL184’s effect. Knockout of CB1Rs in the VTA did not affect morphine preference (*Cnr1*^VTA-KO^ treated with vehicle).

The analgesic response to morphine was not affected by the genotype (fig. S5B). JZL184 pretreatment tends to decrease the ED_50_ values in both genotypes, when compared with their genotype homologous counterparts treated with vehicle, suggesting that deletion of VTA-CB1Rs within the VTA does not inhibit the morphine analgesic response.

### Pharmacologically inhibiting MAGL blunts NAc neural activity and DA neurotransmission

**Fig. 5.**
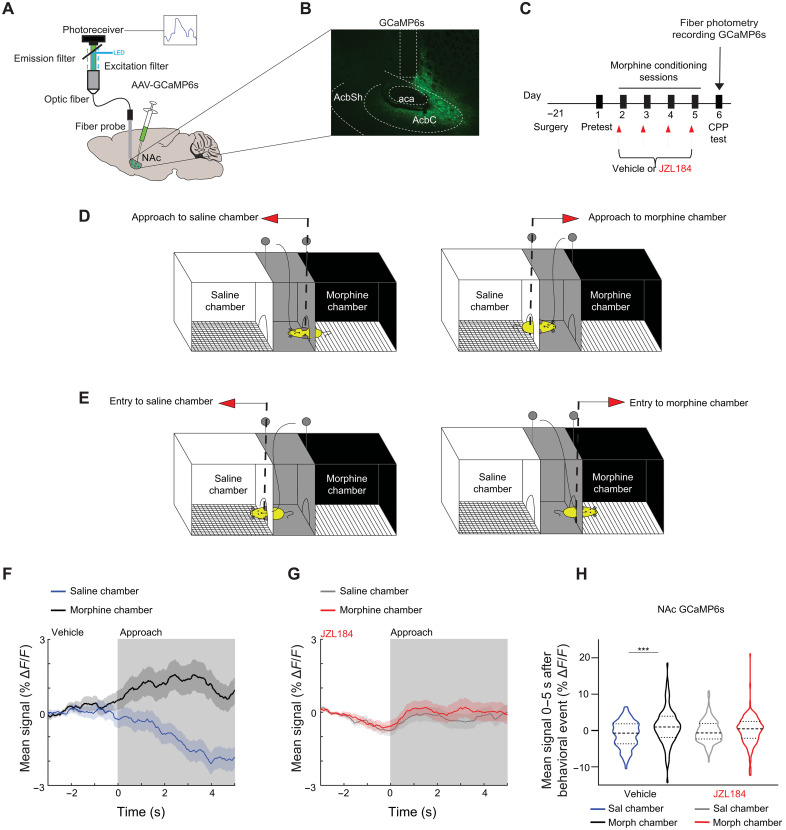
JZL184 attenuates NAc neural activity time-locked to the morphine-paired chamber. (**A**) Brain schematic with an optic fiber in the NAc expressing GCaMP6s and recording apparatus. (**B**) Representative image of GCaMP6s expression in the NAc. aca, anterior commissure; AcbC, NAc core; AcbSh, NAc shell. (**C**) Experimental timeline for surgery, morphine CPP, and FP recording of GCaMP6s. (**D**) Schematic of the CPP box depicting a mouse approaching the saline (left) or the morphine chamber (right). Dashed line represents the position in the CPP box where the FP GCaMP6s or dlight1.2 signal was time-locked to the approach behavior. (**E**) Schematic of the CPP box depicting a mouse entering the saline (left) or the morphine chamber (right). Dashed line represents the entry point in the saline or morphine chamber to which the FP signal was time-locked. (**F** and **G**) Average trace of NAc activity in (F) vehicle-pretreated animals time-locked to the approach of the morphine-paired (black) or the saline-paired chamber (blue) or average trace of NAc activity in (G) JZL184-pretreated animals time-locked to the approach of the morphine-paired (red) or the saline-paired chamber (gray). Gray shading indicates window used for quantification in (H). (**H**) Mean NAc signal during approach (0- to 5-s window) of saline- or morphine-paired chambers in vehicle or JZL184-pretreated animals. In vehicle animals, there was a significant difference in signal during approach of morphine- compared to saline-paired chambers (*N* = 6 mice, 96 morphine chamber approaches, 95 saline chamber approaches; linear mixed effect model ANOVA marginal test, significant effect of group *F*_1,189_ = 12.1; ***P = 0.0006). There was no difference in signal in the JZL184-pretreated animals (*N* = 9 mice, 166 morphine chamber approaches, 163 saline chamber approaches; linear mixed effect model—ANOVA marginal test, no effect of group *F*_1,327_ = 0.37; *P* = 0.54). Error bars ± SEM (number of trials). LED, light-emitting diode.

The VTA plays a critical role in opioid reward (*37*, *38*) and reinforcement (*39*) primarily through activation of dopaminergic afferents projecting to the NAc that increase NAc activity (*40*) via DA neurotransmission. Given this and our behavioral data indicating that enhancing 2-AG blunts opioid reward, we hypothesized that enhancing 2-AG may be preventing the increase in NAc activity and DA release associated with opioid reward. To measure neural activity, we used the genetically encoded Ca^2+^ indicator GCaMP6s alongside fiber photometry (FP) to record in vivo Ca^2+^signals as a proxy for neural activity (*41*–*43*). A virus for GCaMP6s (AAV1-Syn-GCaMP6s-WPRE.SV40) was injected unilaterally into the NAc, and a fiber optic cannula was implanted above the injection site to deliver 470-nm excitation light and record changes in fluorescence during the CPP test (Fig. 5, A to C, and fig. S6A). We quantified activity as animals approached (defined as the time of exit of the previous chamber) and entered the saline- versus morphine-paired chamber (Fig. 5, D and E). Animals with poor viral expression or incorrect fiber targeting were excluded from the analysis. Mice were tested in morphine CPP with vehicle or JZL184 pretreatment during morphine conditioning (as described in Fig. 1B), and FP recordings of NAc activity were obtained during the CPP test session (day 6; Fig. 5C). Vehicle-treated mice showed an increase in NAc GCaMP6s signal as they approached the morphine-paired chamber, while conversely, the signal decreased when approaching the saline-paired chamber (Fig. 5, F and H, and fig. S6B). This difference in signal persisted as mice entered the morphine chamber compared to the saline chamber (Fig. 5E and fig. S6, B, D, and F). In contrast, no change in GCaMP6s signal was seen as mice pretreated with JZL184 approached (Fig. 5, G and H) or entered (fig. S6, C, E, and F) either chamber. Thus, opioid place preference is associated with an elevation in NAc activity as mice approach and enter the opioid-associated chamber, and this activity is fully abolished by JZL184 pretreatment during drug conditioning.

**Fig. 6.**
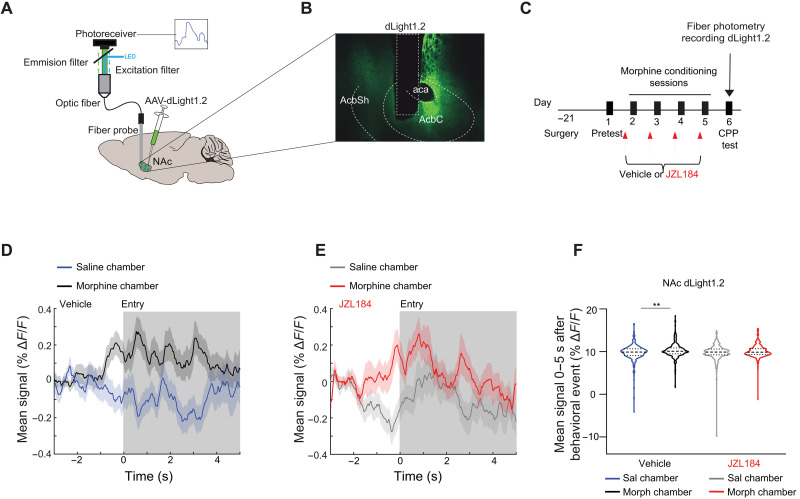
JZL184 pretreatment attenuates NAc DA dynamics time-locked to the morphine-paired chamber. (**A**) Brain schematic with an optic fiber implanted into the NAc expressing dLight1.2 and recording apparatus. (**B**) Representative image of dLight1.2 expression in the NAc. aca, anterior commissure; AcbC, NAc core; AcbSh, NAc shell. (**C**) Experimental timeline for surgery, morphine CPP, and FP recording of DA (dLight1.2) on CPP test day 6. (**D**) Average trace of DA signal in vehicle-pretreated animals time-locked to entry into either the morphine-paired chamber (black) or the saline-paired chamber (blue). Gray shading indicates window used for quantification in (F). *N* = 16 mice, 286 morphine chamber entries, 300 saline chamber entries. (**E**) Average trace of DA signal in JZL184-pretreated animals time-locked to entry into either the morphine-paired chamber (red) or the saline-paired chamber (gray). Gray shading indicates window used for quantification in (F). *N* = 12 mice, 217 morphine chamber entries, 241 saline chamber entries. (**F**) Mean DA signal during entry (0- to 5-s window) of either saline- or morphine-paired chambers in either vehicle or JZL184-pretreated animals. In vehicle animals, there was a significant difference in signal during entry into morphine- compared to saline-paired chambers (*N* = 16 mice, 286 morphine chamber entries, 300 saline chamber entries; linear mixed effect model—ANOVA marginal test, significant effect of group *F*_1,584_ = 8.67; **P = 0.0034. There was no significant difference in signal in the JZL184-pretreated animals (*N* = 12 mice, 217 morphine chamber entries, 241 saline chamber entries; linear mixed effect model—ANOVA marginal test, no effect of group *F*_1,456_ = 2.8; *P* = 0.094). Error bars ± SEM (number of trials).

Next, we used FP to examine DA levels in the NAc using the genetically encoded fluorescent DA sensor, dLight1.2 (*44*). We hypothesized that JZL184 would decrease DA release in the NAc given that it abolished reward-related NAc activity. Mice were injected unilaterally with a viral vector for dLight1.2 (AAV5-hSyn-dLight1.2) into the NAc and implanted with a fiber optic cannula above the injection site to deliver 470-nm excitation light and record changes in fluorescence (Fig. 6, A and B, and fig. S7A). Mice were tested in morphine CPP with vehicle or JZL184 pretreatment during conditioning (as described in Fig. 1B), and FP recordings of DA activity were obtained during the CPP test session (day 6; Fig. 6C). Vehicle-treated mice showed an increase in dLight1.2 signal when they entered the morphine-paired chamber, while the signal decreased as they entered the saline-paired chamber (Fig. 6, D and F, and fig. S7B). This difference in dLight1.2 signal during entry was blunted in JZL184-pretreated mice (Fig. 6, E and F, and fig. S7C). There was no change in signal during approach behavior in vehicle- or JZL184-pretreated mice (fig. S7, D to F). These results are consistent with our hypothesis that elevating 2-AG levels blunts opioid-induced dopaminergic transmission and neural activity within the NAc.

## DISCUSSION

In this study, we demonstrate that systemic and intra-VTA MAGL inhibition with JZL184 [which elevates 2-AG and not AEA; (*45*)] attenuates the rewarding effects of opioids through CB1Rs with no effect on analgesia. In contrast, enhancing AEA has no effect. Our in vivo calcium and DA detection studies indicate that enhancing 2-AG levels diminishes opioid reward–related NAc neural activity and DA transmission. These studies corroborate and expand upon previous pharmacological studies demonstrating a dampening effect of augmented 2-AG on opioid reward (*15*, *17*).

In our study, JZL184 was administered at a relatively low dose (10 mg/kg) with daily administration over a 4-day period, likely leading to a protracted mild enhancement of 2-AG levels, as compared to the acute effects of high doses (18 to 40 mg/kg) of JZL184 previously reported for brain stimulation–based reward behavior (*19*, *46*). In addition, this repeated low dosing schema has previously been shown not to affect CB1R expression or function with regard to antinociceptive properties of the MAGL inhibitor (*47*–*49*). Conversely, repeated high dosing of JZL184 (>16 mg/kg) led to functional down-regulation of CB1R (*49*, *50*). However, repetitive MAGL inhibition with low-dose JZL184 (8 mg/kg, ip) was also recently shown to increase NAc DA levels and facilitate sucrose-based reward (*51*), highlighting the notion that opioid reward–related behavior may be uniquely regulated by the eCB system. This is further supported by our data of the ability of repeated JZL184 to abolish morphine CPP-associated NAc DA levels versus increase in DA associated with brain stimulation and sucrose reward (*19*, *51*). While there is a notable gap in the literature on the impact of elevating the endogenous cannabinoids specifically on opioid reward–related behaviors, a previous study with a dual FAAH-MAGL inhibitor (SA-57) was shown to attenuate heroin self-administration (*15*), with no effect of a FAAH inhibitor (*52*), pointing to the contribution of MAGL inhibition on opioid seeking.

The selective attenuating effects of 2-AG on opioid reward, in our CPP and self-administration paradigms, appear to be specific as our studies indicate that AEA does not alter opioid reward, as measured by CPP. Of note, of these CB1R agonists, 2-AG has the highest apparent affinity and efficacy for the cannabinoid receptor (*53*, *54*). It will be interesting in the future to test whether the relative efficacy of a broader range of CB1R agonists on opioid reward behavior as well as NAc neural activity is dependent on distinct pharmacological properties.

Our studies using VTA-specific knockout of *Cnr1* supports a role for VTA CB1Rs in mediating the effect of JZL184 on blunting opioid preference. However, the precise VTA cell type that is involved remains a question. CB1Rs are expressed by different neurons of the VTA (*55*–*58*), including VTA dopaminergic (*55*, *57*), glutamatergic (*56*, *58*), and GABAergic neurons (*56*, *58*). These cell types have been implicated in driving brain reward (*59*) and thus could be involved in JZL184’s effect. In addition, studies support that opioids mediate rewarding effects by acting on presynaptic MORs on GABAergic inputs onto VTA DA neurons (*60*, *61*). In this model, presynaptic MOR activation disinhibits VTA DA neurons, enabling DA release and increased NAc activity. Once such input is the rostromedial tegmental area (RMTg), also known as the tail of the VTA that provides a major sources of morphine-sensitive inhibitory inputs to the VTA (*62*, *63*), mainly to dopaminergic neurons (*64*). Optogenetic and chemogenetic behavioral studies have strongly implicated this projection in opioid reward (*65*–*67*). CB1Rs are present on these RMTg GABAergic inputs to the VTA (*68*), and therefore, presynaptic CB1Rs within these inputs could be the site of action of JZL184 on morphine reward, suggesting a possible eCB/opioid receptor cross-talk within these GABAergic projection neurons. Further studies are required to identify the specific CB1R-containing cell types within the VTA that mediate the effects of JZL184 as well as test the loss of CB1Rs at GABAergic inputs to the VTA such as those from the RMTg. Notably, eCB activation specifically within VTA neurons or within opioid-sensitive inputs to the VTA may provide an explanation for the specific effects of JZL184 on opioid reward, which is likely mediated further downstream at dopaminergic synapses in the NAc.

Using in vivo calcium and DA FP, we find that JZL184 diminishes opioid reward–related NAc neural activity and DA transmission. More specifically, we found that NAc activity in the vehicle-pretreated control animals was elevated throughout approach and entry into the morphine-paired chamber compared to the saline-paired chamber. Given the observed NAc activity patterns, we initially expected that DA (via dLight1.2) in NAc would show a similar pattern. However, we did not observe similar increases in DA levels during morphine-paired approach, but rather DA levels increased specifically upon entry into the morphine chamber. While unexpected, the relationship between DA release and local cell activity in the NAc is not straightforward, and DA is capable of either exciting or inhibiting NAc neurons, presumably via D1/D2 mechanisms (*69*). One potential explanation for these observed activity patterns is that NAc activity may play a role in “anticipating” the rewarding effects of the morphine-paired chamber (potentially driving the approach behavior), while DA levels are instead critical for responding to and updating (i.e., learning) the perceived value of the chamber upon entry (*70*) with minimal changes occurring during approach.

Although our findings of enhancing 2-AG and its effect on mitigating morphine and oxycodone reward are compelling, it is important to acknowledge certain limitations within our study. While we have demonstrated that enhancing 2-AG attenuates the acquisition of morphine and oxycodone CPP and oxycodone self-administration, we have yet to explore whether enhancing 2-AG similarly affects reinforcing effects of opioids. Future studies will extend our investigation to include other models of opioid reinforcement. In addition, the majority of the studies were performed in male mice, so further studies are needed in female mice to assess the possibility of sex differences. Another area for future studies includes examination of enhancing 2-AG on adverse effects of opioids such as withdrawal and respiratory depression. It is worth noting that a dual MAGL and FAAH inhibitor mitigates opioid-induced withdrawal symptoms (*71*). Here, we report that the effects of JZL184 on opioid reward depend on CB1Rs within the VTA. Recent studies have identified the presence of CB1Rs in different cell types of the VTA (*55*, *56*, *58*). Future experiments will explore the cell type specificity of JZL184’s effect. As next steps, we will pursue studies to define the circuity and molecular mechanisms involved in 2-AG–induced blunting of opioid reward. With regard to the analgesic response, our studies demonstrated that systemic and intra-VTA JZL184 does not inhibit the analgesic response of opioids, as determined using the tail-flick test and the hot plate test. While these two assays primarily assess spinal reflexes, several studies demonstrate that intra-VTA injections of various compounds modify the tail-flick test response (*72*–*76*), indicating that the brain, particularly the VTA, plays a role in this alteration. In addition, other studies have shown the importance of additional brain regions in mediating opioid’s analgesic response using the hot plate test (*77*, *78*). Further studies are needed using additional analgesia and pain assays to further investigate the role of 2-AG–dependent signaling within specific brain circuitry in mediating the analgesic effects of opioids.

In sum, we have identified a functional interaction between the opioid and eCB systems, by which elevation of the eCB 2-AG leads to attenuated opioid-mediated reward. These findings highlight an unexpected role of 2-AG in regulating reward processes that is distinct from other cannabinoid ligands. In addition, the present findings provide a compelling rationale for developing a class of adjunctive eCB-based treatments that could dissociate the rewarding and analgesic properties of opioids.

## MATERIALS AND METHODS

### Animals

C57BL/6 male mice (Charles River Laboratories) or C57BL/6J female and male mice (the Jackson Laboratory) were 8 to 10 weeks of age at the start of the experiments. Mice were provided with food and water ad libitum. Animals were kept on a 12-hour light/dark cycle (from 7 a.m. to 7 p.m.). *Cnr1* floxed mice on C57BL/6 background were obtained from J. Elmquist and T. Fujikawa of University of Texas Southwestern Medical Center.

### Drugs and inhibitors

Morphine was obtained through the National Institute on Drug Abuse following the Ordering Guidelines for Research Chemicals and Controlled Substances. Cocaine HCl and oxycodone HCl were purchased from Sigma-Aldrich (St. Louis, MO). The FAAH inhibitor PF-3485 and the MAGL inhibitor JZL184 were obtained from Cayman Chemical. PF-3845 and JZL184 were dissolved in dimethyl sulfoxide (DMSO) and diluted to their respective final concentrations in a solution of DMSO, Tween 80, and saline at a ratio of 10:10:80.

### Conditioned place preference

As previously published, opioid (*36*) CPP was performed using a three-chamber (black, white, and a center gray chamber) apparatus (Med Associates Inc.). We use a 6-day protocol with 4 days of conditioning. Briefly, on day 1 (pretest), mice were placed in the central gray chamber for 1 min of habituation followed by free exploration in the three chambers for 20 (morphine CPP) or 30 (oxycodone CPP) min. Time spent in the black and white chamber was recorded. During conditioning days (days 2 to 5), in the morning session, mice were injected with saline (ip; 0.01 ml/g body weight) and confined to the chamber they preferred during pretest for 20 or 30 min. During the afternoon, mice were injected with morphine (10 mg/kg, ip) or oxycodone (1 mg/kg, ip) and confined to the opposite chamber for 20 min (morphine) or 30 min (oxycodone). On day 6 (CPP test), mice were placed in the central gray chamber for 1 min of habituation followed by free exploration in the three chambers 20 (morphine CPP) or 30 (oxycodone CPP) min. For pharmacological experiments, mice were pretreated with JZL184 (10 mg/kg) and PF-3845 (10 mg/kg) (*28*, *79*) or vehicle (10% DMSO and 10% Tween 80 in saline) for 2 hours before morphine or oxycodone treatment (*26*, *27*). Preference score was calculated as time spent in the opioid-paired chamber minus time spent in the saline-paired chamber. Mice are defined as having acquired morphine or oxycodone place preference when the preference score on the CPP test session (day 6) is significantly higher than the preference score on the pretest (day 1). Locomotor activity (based on total beam breaks) was obtained on the test day while mice were in the CPP chambers.

### Jugular catheterization surgery

For self-administration studies, animals underwent jugular catheterization. Before surgery, mice were anesthetized with ketamine (80 mg/kg) and xylazine (12 mg/kg). An indwelling silastic catheter was placed into the right jugular vein and sutured in place. The catheter was then threaded subcutaneously over the shoulder blade and was routed to a mesh back mount platform (Instech Laboratories Inc.) that secured the placement. Catheters were flushed daily with 0.1 ml of an antibiotic (Timentin, 0.93 mg/ml) dissolved in heparinized saline.

### Operant food training

Three days following catheterization, mice were trained to perform an operant response for sucrose pellets. Mice were placed in operant chambers (Med Associates) and trained to press a lever to receive a sucrose pellet. Mice performed two consecutive days of FR1 responding. A compound cue stimulus consisting of a cue light above the active lever, a 2900-Hz tone, and house light off was concurrent with each pellet administration, followed by an additional 8-s time-out when responding had no programmed consequences and the house light remained off. Mice were allowed to self-administer a maximum of 50 pellets per self-administration session. During the food training phase, mice were food restricted to >90% of their free-feeding weight. Mice returned to ad libitum food access 5 days following the start of oxycodone self-administration.

### Oxycodone-self administration

Mice were tested for oxycodone self-administration behavior in 2-hour sessions in the same chamber used for sucrose pellet self-administration. Animals were randomly assigned to receive either JZL184 (10 mg/kg, ip) or vehicle (10% DMSO and 10% Tween 80 in saline or saline alone; there was no significant difference in behavior between these two control groups, and thus, they were combined; fig. S2, C and D) 2 hours before the start of each of the oxycodone self-administration sessions (wheels and levers). Mice were first tested on three consecutive days of oxycodone self-administration using a wheel. Mice were trained to spin a wheel quarter of a turn that delivered an intravenous oxycodone injection (0.25 mg/kg per infusion). During testing, responding on the wheel delivered an intravenous oxycodone infusion (0.25mg/kg per infusion), paired with the same compound cue under the same schedule as the food training, followed by an 8-s time-out when responding had no programmed consequences. Following 3 days of wheels, mice were transitioned from a wheel back to a lever, which they had previously learned to press during the operant food training phase. Press of an active lever delivered an intravenous oxycodone injection (0.25 mg/kg per infusion), whereas press of an inactive did not have any consequences. Mice were evaluated for oxycodone self-administration on an FR1 schedule for an additional 7 days. The percent of active lever presses during the operant sessions was calculated using the formula (number of active lever press)/(number of active lever press + number of inactive lever press) × 100.

### Hot plate assay

Mice were tested for analgesia using the hot/cold plate apparatus (UGO Basile). First, mice were tested for their baseline response by placing animals on the platform of the hot plate set at 55°C. The time(s) elapsing to the first pain response (licking or jumping) was scored. Then, mice were injected with JZL184 (10 mg/kg, ip) or vehicle, and 2 hours later, mice were tested for morphine analgesia with a cumulative dose-response curve (0.3 to 10 mg/kg), using the hot plate. Mice were tested 30 min after each dose of morphine was administered. A maximal latency of 30 s was used to minimize any tissue damage. Results were determined as the percentage of maximum possible effect (%MPE) [(latency after drug − baseline latency)/(30 − baseline latency) * 100].

### Tail-flick test

Mice were tested for analgesia using the Ugo Basile 37360 tail-flick device. For the baseline test, mice were wrapped, and the lower one-third of the tail was placed over the sensor of the device that emitted radiant heat. The animals’ response to the heat is a flick of the tail. The latency of the tail flick was obtained as a measure of the baseline response with a maximal latency of 10 s to minimize tissue damage. Then, mice were pretreated with JZL184 (10 mg/kg, ip) or PF-3845 (10 mg/kg, ip), and 2 hours later, mice were tested for morphine analgesia with a cumulated dose-response curve (0.1 to 10 mg/kg), or for oxycodone analgesia with a cumulated dose-response curve (0.1 to 5 mg/kg), doses were given every 30 min. The maximum time the tail is exposed to the tail-flick device is 10 s to avoid severe burns. Results were calculated as the % MPE as follows: [(latency after drug − baseline latency)/(10 − baseline latency) × 100].

### Elevated plus maze

Elevated plus maze testing was performed as previously reported (*80*). Briefly, mice were placed in the center of an elevated cross-shaped maze. Behavior was video recorded for 5 min and analyzed using the ANY-maze software. Data are reported as the percent of time spent in the open arms which was calculated as [time in open arms (s)/total time (s)] × 100.

### Tail suspension test

Tail suspension test (TST) was performed as previously published (*81*). Briefly, TST was performed by suspending a mouse 30 cm from the floor using a 17-cm-long adhesive tape which was secured to the tail 2 cm from the tip. Each mouse was video recorded for 6 min, and time spent immobile was hand-scored by an experimenter blinded to the treatments using the computer-assisted software CowLog.

### Intracranial surgery

For the delivery of JZL184 or vehicle into the VTA, guide cannulas were implanted bilaterally in adult mice as previously published (*82*). The coordinates were anterior/posterior (AP) = 0.4 to 0.7 mm, medial/lateral (ML) = −3.1 to −3.3 mm, and dorsal/ventral (DV) = 4.2 to 4.4 mm (using Paxinos and Franklin’s the Mouse Brain in Stereotaxic Coordinates atlas as a reference). A total of 5 μg/μl, 200 nl/side per min of JZL184 was bilaterally microinjected 30 min before each morphine conditioning session (*83*). After the completion of behavioral experiments, accurate guide cannula placement was confirmed by Nissl staining. All the cannula placements were mapped using the Paxinos and Franklin’s the Mouse Brain in Stereotaxic Coordinates atlas (fig. S4A). Mice with improper cannula placement were excluded from analyses.

For in vivo FP experiments, surgical procedures were conducted as previously published (*43*). A viral vector expressing GCaMP6s (AAV1-Syn-GCaMP6s- WPRE.SV40, from Addgene), or dLight1.2 (AAV5-hSyn-dLight1.2, from Addgene), was injected unilaterally into the NAc. The coordinates were 1.0 to1.2 mm AP relative to bregma, 0.75 to 0.85 mm ML, and −4.5 to −4.75 DV (using Paxinos and Franklin’s the Mouse Brain in Stereotaxic Coordinates atlas as a reference). A total of 250 nl of GCaMP6s or 600 to 800 nl of dLight1.2 at a rate of 50 nl/min was injected unilaterally. In addition, during the same surgery, a 400-μm-diameter optical fiber (Doric) was implanted approximately 0.2 mm dorsal of the virus injection site and was secured with Metabond. Following surgery, mice were housed with ad libitum access to food and water and were allowed a minimum of 21 days for viral expression before all experiments. After the completion of behavioral experiments, accurate virus injection and fiber cannula placement was confirmed by immunofluorescence. All the fiber placements and viral expressions were mapped using Paxinos and Franklin’s the Mouse Brain in Stereotaxic Coordinates atlas (figs. S5A and S6A). Mice with improper virus injection or fiber placement were excluded from FP analyses.

For conditional knockout of *Cnr1*, AAV2-GFP-Cre (Vector Biolabs) was bilaterally injected into the VTA of *Cnr1* wild-type (*Cnr1*^WT^) or *Cnr1* floxed (*Cnr1*^fl/fl^) mice (*84*)*.* The coordinates used to inject the virus were −3.1 mm AP relative to bregma, 0.4 mm ML, and −4.4 DV (using Paxinos and Franklin’s the Mouse Brain in Stereotaxic Coordinates atlas as a reference). A total of 300 nl of AAV2-GFP-Cre, at a rate of 100 nl/min, was injected bilaterally. Following surgery, mice were housed with ad libitum access to food and water and were allowed a minimum of 21 days for viral expression before all experiments. After the completion of all the behavioral experiments, accurate virus injection was confirmed by immunofluorescence, and focal knockout of *Cnr1* within the VTA was confirmed with RNAscope. All the viral expressions in *Cnr1*^fl/fl^ mice were mapped using Paxinos and Franklin’s the Mouse Brain in Stereotaxic Coordinates atlas (fig. S5A). Mice with improper injections were excluded from statistical analyses.

### In vivo FP recording

In vivo FP was performed to measure calcium-dependent activity and DA levels during CPP behavior as previously described (*43*). Briefly, 2 to 3 days before behavioral testing, mice were habituated to a patch cord attached to the implanted optical fiber for at least 1 min in the animal’s home cage. The patch cord is long enough to allow the animal to move freely across the three chambers of the CPP apparatus. The three-chamber apparatus has two sliding doors that separate the center chamber from the other two chambers. The sliding doors are removed for facilitation of the animal movement with the patch cord. However, the chambers remain distinct, allowing for clear determination of the animals’ location. During morphine CPP test (day 6), FP recordings were taken during the 20-min CPP test. In addition, the behavioral test was video recorded by a camera above the CPP apparatus. Animals found to have poor viral expression, incorrect fiber placement during histological verification, or identified outliers were excluded before analysis, and no further animals were excluded during analysis. Data were analyzed using MATLAB (MathWorks). Raw fluorescent signal was normalized and transformed to Δ*F*/*F* by taking the median value of the overall trace, subtracting this median value from all data points, and then dividing by the median. The NAc GCaMP6s and dLight1.2 fluorescent signals were time-locked to hand-scored morphine CPP behavior. Experimenter was blinded to the treatment while hand-scoring. Chamber entry activity was analyzed by time-locking signal to the hand-scored times at which animals entered either the saline- or morphine-paired chamber. Chamber approach was analyzed by time-locking signal to the hand-scored time at which animals exited either the saline-paired (with next chamber entry into morphine paired) or morphine-paired (with next chamber entry into saline paired) chambers. Thus, if an animal exited the saline-paired chamber but proceeded to reenter the saline chamber, then this was not considered an approach behavior. To quantify the activity levels related to entry and approach behaviors, the average signal for a window 0 to 5 s from behavior onset was taken. Average photometry traces are represented as the mean ±95% confidence interval (CI). Statistical analysis of photometry data used a linear mixed effects model in which mouse identity was modeled as a random effect to account for repeated measures from each mouse.

### Histology

After completion of behavioral tests, animals were transcardially perfused with 0.1 M phosphate buffer (PB) followed by 4% paraformaldehyde (pH 7.4) as previously described (*43*). Nissl staining for guide cannula placements or GFP immunofluorescence was performed for GCaMP6s, dLight1.2, and Cre in *Cnr1* floxed mouse for viral placement as previously described (*43*). Briefly, sections were washed twice for 5 min in 0.1 M PB with 0.3% Triton X-100 buffer. Next, sections were incubated in blocking solution (0.1 M PB with 1% normal goat serum and 1% bovine serum albumin) for 30 min at room temperature followed by incubation with the primary antibody chicken anti-GFP (1:2000; Aves Laboratories) in blocking solution for 1 hour at room temperature. Sections then were washed twice for 5 min in 0.1 M PB, followed by incubation with Alexa Fluor 488 goat anti-chicken immunoglobulin G secondary antibody in 0.1 M PB with 0.3% Triton X-100 for 30 min at room temperature. Slides were cover-slipped using Prolong Gold containing 4′,6-diamidino-2-phenylindole (DAPI; Thermo Fisher Scientific).

### RNAscope combined with immunofluorescence

Paraformaldehyde fixed brains were sectioned at 30 μm on a Leica vibratome, and VTA-containing brain slices were chosen on the basis of the Paxinos and Franklin’s the Mouse Brain in Stereotaxic Coordinates atlas. To confirm viral expression, all VTA-containing brain slices from each animal were temporarily mounted on a slide using 1× phosphate-buffered saline (PBS) and visualized under a Nikon Ni-E upright fluorescence microscope in the GFP channel at ×20 magnification. Two VTA-containing brain sections from each animal were chosen on the basis of the most intense GFP expression, in a blinded fashion to the animal experimental group (total of six animals) and mounted on charged slides using 1× PBS, and then allowed to air dry overnight. The next day, the mounted tissue was baked in a HybEZ oven (ACDBio) at 60°C for 1 hour. The sections were then acclimated for 10 s in near-boiling deionized water (dH_2_O) and incubated in 1× target retrieval buffer (catalog no. 322000, ACDBio) (at a temperature of 98° to 102°C) for 5 min, followed by three washes using room temperature dH_2_O. The sections were then submerged in 100% ethanol for 3 min and allowed to air dry, followed by two washes with dH_2_O. All subsequent incubation periods were performed at 40°C and washes for 2 min each. The sections were then incubated in Protease III (catalog no. 322337, ACDBio) for 30 min, washed four times with dH_2_O, followed by incubation with either a positive control probe (catalog no. 322881, ACDBio), negative control probe (catalog no. 3220871, ACDBio), or *Cnr1*-C2 probe (catalog no. 420721, probe diluent catalog no. 300041, ACDBio) for 2 hours. Slides were then washed in 1× wash buffer (catalog no. 310091, ACDBio). Sections were incubated for 30 min with AMP1 (catalog no. 323101, ACDBio), AMP2 (catalog no. 323102, ACDBio) for 30 min, and AMP3 (catalog no. 323103, ACDBio) for 15 min, with one wash with 1× wash buffer performed in-between the previous incubation steps. Next, sections were incubated in horseradish peroxidase (HRP) C2 (catalog no. 323105, ACDBio) for 15 min, followed by two washes with 1× wash buffer. Then, sections were incubated with trichostatin A (TSA) Vivid fluorophore 570 (catalog no. 323272, ACDBio) in TSA buffer (catalog no. 322809, ACDBio) for 30 min, followed by incubation with HRP blocker (catalog no. 323107, ACDBio) for 15 min, and two subsequent washes. To identify colocalization of GFP-tagged virus with *Cnr1* mRNA, immunofluorescence for GFP was conducted following RNAscope (as described above).

For imaging and quantification of RNAscope measurements, experimenter was blinded to animal groups. Images were taken as a z-stack, with bidirectional X and four-frame averaging using a confocal microscope. All channels, laser intensities, gains, and detection ranges were kept consistent between scans, and all scans were taken during the same confocal microscopy session to avoid changes in tissue quality. GFP channel (488 laser; excitation, 488; emission, 525; with detection range of 500 to 550) was used to visualize GFP-positive cells, and DSRed (561 laser; excitation, 561; emission, 603; detection range, 580 to 625) was used to visualize the presence of the TSA Vivid Dye 570 that detected *Cnr1* puncta. After acquisition at ×40 and ×20 magnifications, images were processed for quantification using Fiji/ImageJ. One z-stack from each scan was chosen and placed through automatic thresholding of the GFP channel. Using Analyze Particles, automatic region of interest (ROI) was made around the soma of each GFP- expressing cell and added to the ROI manager. In parallel, automatic ROIs were created for the background to use as a normalizer for the corrected total cell fluorescence (CTCF), which was calculated as CTCF = integrated density − (area of selected cell X mean fluorescence of background readings). The area, integrated density, and mean gray values were calculated for all set ROIs. The CTCF was used to confirm that neurons chosen were expressing substantial levels of fluorescence when normalize to the background. Further, confirmation of GFP expression by cells was confirmed with DAPI staining. For the DSRed channel, the scans showed brightly lit foci, which were TSA Vivid Dye 570–labeled *Cnr1* puncta. Similar to the GFP channel, automatic thresholding was used as well as the Gaussian Blur and Subtract Background processes to remove any nonspecific labeling. This was followed by using Find Maxima in ImageJ, with the output type set to “point selection” and with the selection set to “above lower threshold” in the dialog box. The DSRed channel was layered over the previously found GFP ROIs, and after using Analyze-Measure, a total number of puncta for the *Cnr1* probe was gathered within each RO1 (cell). Once data were collected, experimenter was unblinded to the experimenter group. Forty-two cells in total were counted across three animals for control *Cnr1* VTA-WT animals and 25 cells in total were counted across three animals for *Cnr1*^VTA-KO^ animals. The total number of puncta, which reflects the total number of *Cnr1* transcripts per GFP-positive cell, was used for comparison across experimental groups.

### Statistical analysis

GraphPad Prism 8.0 was used for the analyses of the behavioral tests, while MATLAB with custom scripts was used for the analyses of the in vivo FP recordings. Before performing statistical analyses, outliers were identified from each of the CPP (using preference score data on pretest test and test days) and self-administration (using the number of infusions on each behavioral testing day) experiments using the two SD rule (any data point more than two SDs above or below the mean). For any outliers identified, all data for the animal were removed from the subsequent statistical analyses. For all the CPP behavioral data (except for Figs. 1G and 4H), a two-way analysis of variance (ANOVA) statistical analysis was performed to account for the treatment (vehicle or JZL184) and the day/session (pretest or test), followed by a Bonferroni post hoc test. For the CPP experiment presented in Fig. 1G, a three-way ANOVA was performed to account for CB1 inverse agonist pretreatment (vehicle or AM251), treatment (vehicle or JZL184), and day/session (pretest or test), followed by a Bonferroni post hoc test. This was performed for each of the doses of AM251 used. For the CPP experiment presented on Fig. 4H, a three-way ANOVA was performed to account for genotype (*Cnr1*^VTA-WT^ or *Cnr1*^VTA-KO^), treatment (vehicle or JZL184), and day/session (pretest or test), followed by a Bonferroni post hoc test. For the oxycodone self-administration experiments, a two-way Repeated Measures (RM) ANOVA to account for treatment (vehicle or JZL184) and days (1 to 10). For the average of infusions, quantification of puncta, locomotor activity, time in open arms, immobility time, sucrose pellet consumption, and the average of % active lever presses an unpaired *t* test was performed. For the analgesia tests, the ED_50_ values with 95% CIs (parentheses) were determined by nonlinear regression analysis, ED_50_ values with nonoverlapping 95% CIs were considered significantly different, but we also performed a two-way ANOVA to account for treatment (vehicle or JZL184) and doses or three-way RM ANOVA (fig. S3, D and F) to account for treatment (vehicle or JZL184), time (pretreatment or posttreatment), and doses. For fig. S3H, a two-way RM ANOVA per day was performed, to account for treatment (vehicle or JZL184) and time (30, 60, 90, or 120 min). For analysis of the FP recordings, MATLAB was used to perform a mixed linear effects model to compare an average signal amplitude (Δ*F*/*F*) of 0 to 5 s while approaching the chamber or upon entry to the respective chambers, with the animal modeled as a random effect and the CPP chamber (drug versus saline) modeled as a fixed effect. Exact sample sizes are indicated in figure legends for each experiment.

### Study approval

All procedures were conducted in accordance with the National Institutes of Health guidelines for the Care and Use of Laboratory Animals, and animal use was reviewed and approved by the Weill Cornell Medicine Institutional Animal Care and Use Committee (IACUC) (protocol nos. 2010-0004 and 0605-491A) and Temple University IACUC (protocol nos. 5182 and 5189).
