# Supplementary material for: Elevating levels of the endocannabinoid 2-arachidonoylglycerol blunts opioid reward but not analgesia
Source: Sci Adv. 2024 Nov 29;10(48):eadq4779. doi: 10.1126/sciadv.adq4779 (PMC11606496; doi:10.1126/sciadv.adq4779)
Supplement: Supplementary file 1 — Figs. S1 to S7 [file sciadv.adq4779_sm.pdf]

Science Advances
AAAS

Supplementary Materials for

Elevating levels of the endocannabinoid 2-arachidonoylglycerol blunts opioid reward but not analgesia

Arlene Martínez-Rivera *et al.*

Corresponding author: Francis S. Lee, fslee@med.cornell.edu;
Anjali M. Rajadhyaksha, anjali.rajadhyaksha@temple.edu

Sci. Adv. **10**, eadq4779 (2024)
DOI: 10.1126/sciadv.adq4779

This PDF file includes:

Figs. S1 to S7

Supplementary Fig. 1

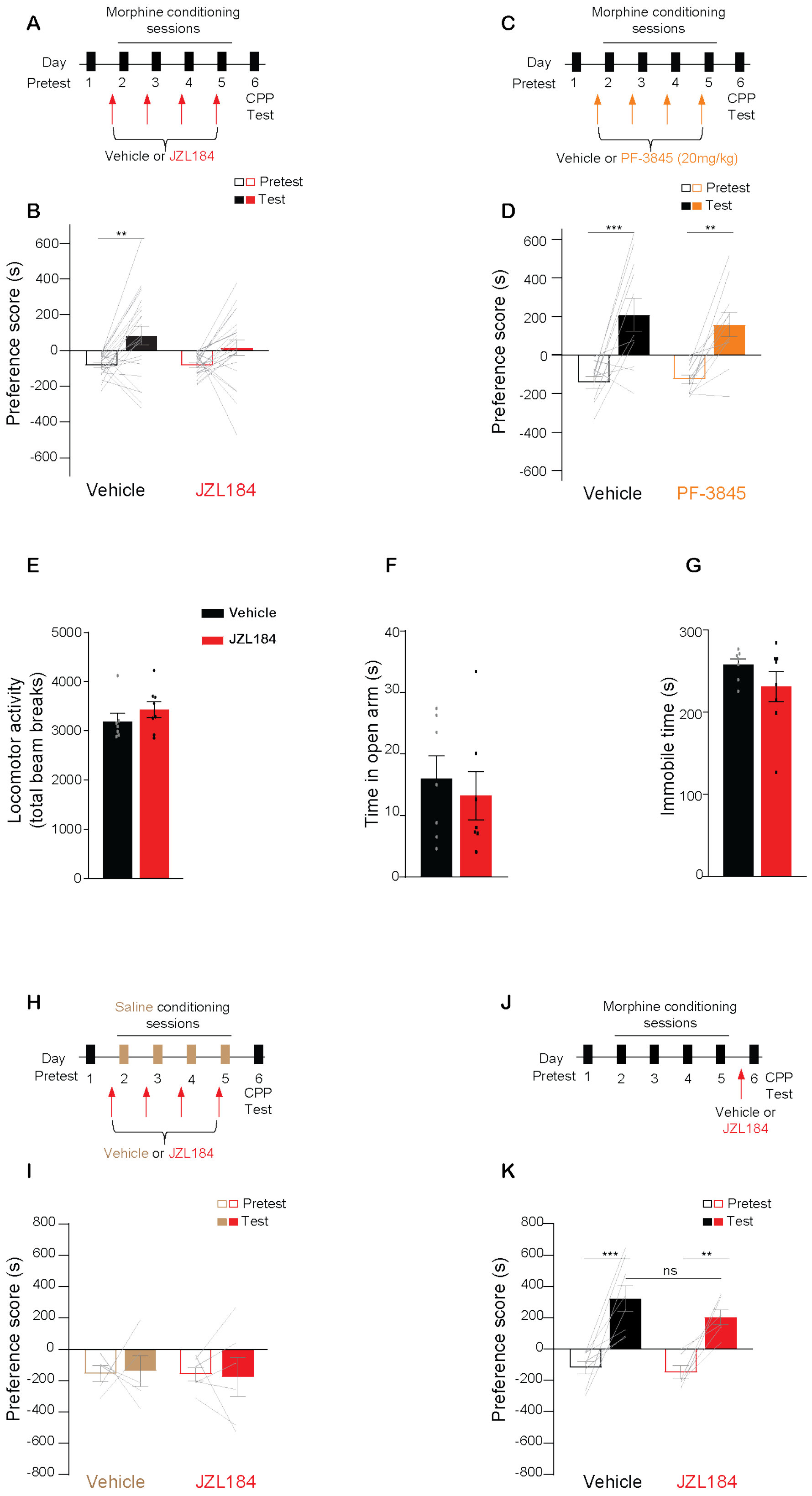

Fig. S1. JZL184 attenuates morphine CPP in female mice, PF-3845 has no effect on morphine CPP in male mice, and JZL184 has no effect on the expression of morphine CPP, locomotion, EPM, and TFT. (**A**) Timeline of behavioral protocol for morphine CPP and JZL184 (10mg/kg) systemic injections pretreatment in female mice. (**B**) Systemic injection of JZL184 prior to each morphine conditioning session abolished morphine CPP (Two-way ANOVA, main effect of day, $F_{1,84} = 0.95$, $P < 0.0001$; Post hoc: Vehicle: Test vs Pretest $^{**}P = 0.002$, JZL184: Test vs Pretest $P = 0.095$, Vehicle $N = 22$, JZL184 $N = 22$). (**C**) Timeline of behavioral protocol for morphine CPP and PF-3845 (20mg/kg i.p.) systemic injections pretreatment. (**D**) Systemic injection of PF-3845 (20mg/kg i.p.) had no effect on morphine CPP (Two-way ANOVA, main effect of day, $F_{1,40} = 31.79$, $P < 0.0001$; Post hoc: Vehicle: Test vs Pretest $^{***}P = 0.0002$, PF-3845: Test vs Pretest $^{**}P = 0.0019$, Vehicle $N = 11$, PF-3845 $N = 11$). (**E**) Locomotor activity on morphine CPP test day is not significantly different between vehicle and JZL184 pretreated mice (Ttest, $t_{(13)} = 1.056$, $P = 0.3101$, Vehicle $N = 7$, JZL184 $N = 8$). (**F-G**) Time in the open arm of the elevated plus maze (Ttest, $t_{(12)} = .5205$, $P = 0.6122$, Vehicle $N = 7$, JZL184 $N = 7$). (**F**) and immobile time in the tail suspension test (**G**) is not significantly different between vehicle and JZL184 pretreated mice (Ttest, $t_{(13)} = 1.296$, $P = 0.2176$, Vehicle $N = 5$, JZL184 $N = 6$), (**H**) Timeline of behavioral protocol for JZL184 on its own during CPP. (**I**) Systemic JZL184 on its own has no effect on conditioning place preference. (Two-way ANOVA, no main effect of day, $F_{1,18} = 3.450^{-006}$, $P = 0.9985$, or treatment $F_{1,18} = 0.0505$, $P = 0.8247$ Vehicle $N = 5$, JZL184 $N = 6$). (**J**) Timeline of behavioral protocol for morphine CPP and systemic injection of JZL184 before the conditioning test. (**K**) Male mice pretreated with systemic injection of JZL184 prior to expression test exhibited morphine CPP similar to vehicle pretreated mice (Two-way ANOVA, main effect of day, $F_{1,24} = 44.82$, $P = 0.0001$; Post hoc: Vehicle: Test vs Pretest $^{***}P < 0.001$, JZL184: Test vs Pretest $^{**}P < 0.0038$, Vehicle $N = 8$, JZL184 $N = 6$). Error bars ± SEM.

Supplementary Figure 2

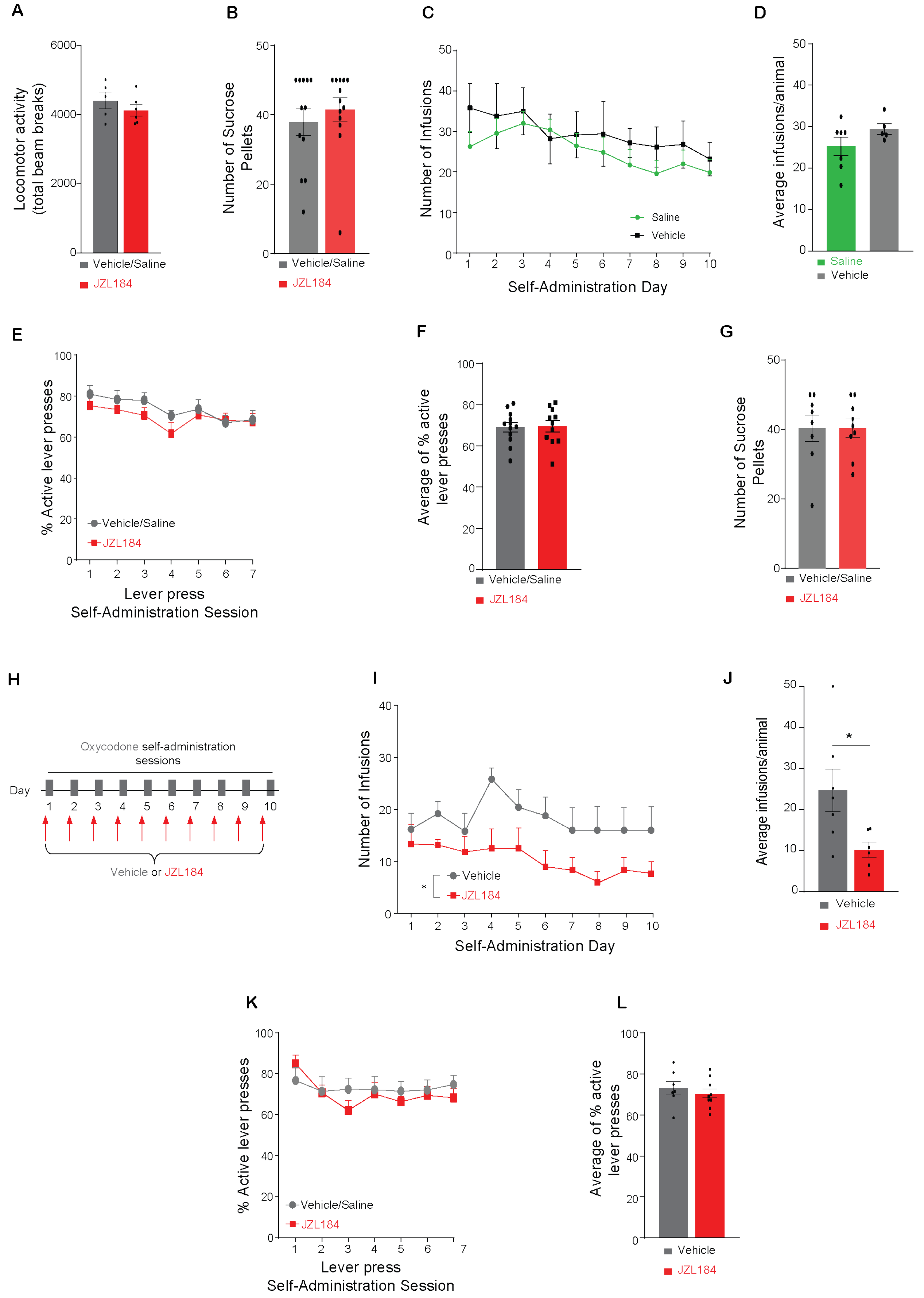

Fig. S2. JZL184 pretreatment does not alter locomotor activity or the percentage of correct choices during oxycodone self-administration and JZL184 attenuates oxycodone self-administration in female mice. (**A**) Locomotor activity on oxycodone CPP test day is not significantly different between vehicle and JZL184 pretreated mice (Ttest, $t_{(9)}$ =0.9812, *P* = 0.3521, Vehicle *N* = 5, JZL184 *N* = 5). (**B**) Mice were trained in the operant self-administration apparatus with sucrose pellets, then they were assigned to receive JZL184 or Vehicle/Saline, no differences between the groups were detected during the self-administration of sucrose pellets (Ttest, $t_{((23)}$ = 0.7050, *P* = 0.4879, Vehicle *N* = 12, JZL184 *N* = 13). (**C-D**) There was no significant difference in systemic Saline or Vehicle (saline, DMSO and Tween80) treatment prior to oxycodone self-administration sessions. (**C**; Two-way RM-ANOVA demonstrated no main effect of treatment, $F_{1, 10}$ = 2.177, *P* = 0.1709; **D**; Average of total infusions/animal, Ttest, $t_{(10)}$ = 1.475, *P* = 0.1709, Saline N = 7, Vehicle N = 5). Error bars ± SEM. (**E-F**) Percentage of active lever presses during oxycodone self-administration does not differ between JZL184 or Vehicle/Saline across days (**E;** Two Way-RM ANOVA, main effect of days, $F_{3.893, 85.64}$ = 3.847, *P* = 0.068, no main effect of treatment, $F_{1, 22}$ = 1.144, *P* = 0.2965) or across animals (**F;** Ttest, $t_{(22)}$ = 1.069, *P* = 0.2965, Vehicle *N* = 12, JZL184 *N* = 12). (**G**) Female mice were trained in the operant self-administration apparatus with sucrose pellets, then they were assigned to receive JZL184 or Vehicle, no differences between the groups were detected during the self-administration of sucrose pellets (Ttest, $t_{(15)}$ = 0.0151, *P* = 0.9881, Vehicle *N* = 8, JZL184 *N* = 9). (**H**) Timeline of behavioral protocol for oxycodone self-administration and JZL184 systemic injections pretreatment. (**I-J**) Systemic JZL184 exposure prior to oxycodone self-administration sessions, attenuated the intake of oxycodone (**I;** Two Way-RM ANOVA, main effect of JZL184 treatment, $F_{1,14}$ = 5.960, **P* = 0.029, main effect of days, $F_{2.482,34.75}$ = 11.01, **P* < 0.0270), **J;** Average of total infusions/animal (Ttest, $t_{(11)}$ = 2.477, **P* = 0.031, Vehicle *N* = 7, JZL184 *N* = 6). (**K-L**) Percentage of active lever presses during oxycodone self-administration in females does not differ between JZL184 or Vehicle across days (**K**; Two Way-RM ANOVA, no main effect of treatment, $F_{1,14}$ = 0.4817, *P* = 0.499, no main effect of days, $F_{3.789, 53.05}$ = 1.865, *P* = 0.13360) or across animals; **L**; Ttest, $t_{(14)}$ = 0.6940, *P* = 0.4990, Vehicle *N* = 7, JZL184 *N* = 9). Error bars ± SEM.

Supplementary Fig. 3

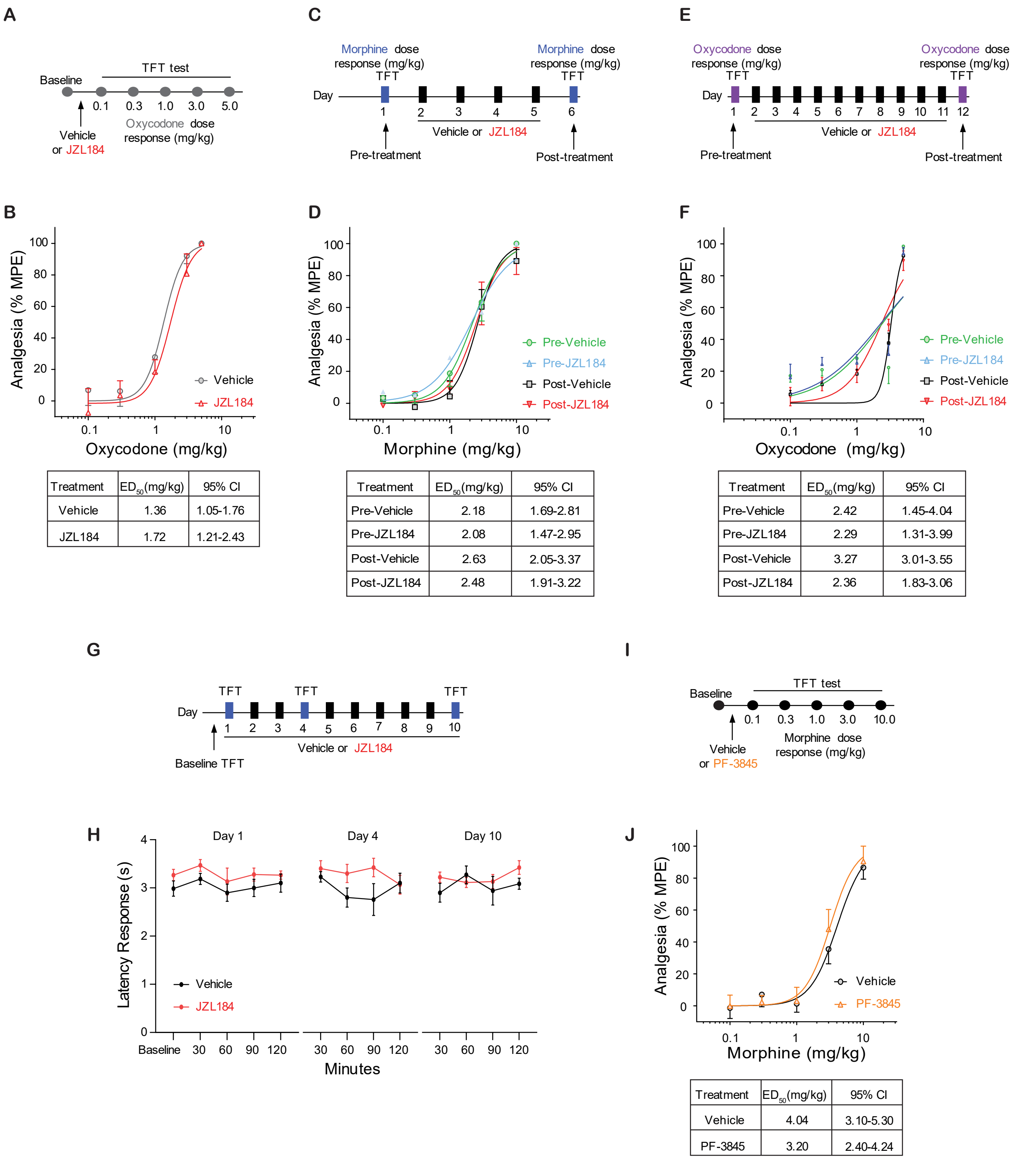

Treatment	ED_{50}(mg/kg)	95% CI
Vehicle	1.36	1.05-1.76
JZL184	1.72	1.21-2.43

Treatment	ED_{50}(mg/kg)	95% CI
Pre-Vehicle	2.18	1.69-2.81
Pre-JZL184	2.08	1.47-2.95
Post-Vehicle	2.63	2.05-3.37
Post-JZL184	2.48	1.91-3.22

Treatment	ED_{50}(mg/kg)	95% CI
Pre-Vehicle	2.42	1.45-4.04
Pre-JZL184	2.29	1.31-3.99
Post-Vehicle	3.27	3.01-3.55
Post-JZL184	2.36	1.83-3.06

Treatment	ED_{50}(mg/kg)	95% CI
Vehicle	4.04	3.10-5.30
PF-3845	3.20	2.40-4.24

Fig. S3. JZL184 and PF-3845 do not inhibit opioid analgesia. (**A**) Experimental timeline of acute systemic vehicle or JZL184 pretreatment prior to the oxycodone dose response in the tail-flick test. (**B**) JZL184 pretreatment has no effect on oxycodone-induced analgesia (Two-way ANOVA, main effect of dose, $F_{4, 55} = 53.44$, $P < 0.001$ Vehicle $N = 7$, JZL184 $N = 6$). (**C**) Experimental timeline of

tail-flick test to measure morphine analgesia pre and post repeated 4 days of vehicle or JZL184 treatment. (**D**) Repeated JZL184 does not inhibit morphine analgesia (Three-way RM ANOVA, main effect of dose, $F_{4,52} = 104.4$, $P < 0.001$. Vehicle $N = 7$, JZL184 $N = 8$). (**E**) Experimental timeline of tail-flick test to measure morphine analgesia pre- and post-repeated 10 days of vehicle or JZL184 treatment. (**F**) Repeated JZL184 does not inhibit morphine analgesia (Three-way RM ANOVA, significant interaction of dose x day, $F_{4, 78} = 6.251$, $P = 0.0002$, and a main effect of dose, $F_{4, 78} = 194.8$, $P < 0.0001$. Vehicle $N = 7$, JZL184 $N = 5$). (**G**) Experimental timeline of tail-flick test to measure if JZL184 induce any analgesic response (**H**) There is no difference in the latency response between vehicle or JZL184 treated mice at any day or time tested (Two-way RM ANOVA on Day 1, no main effect of treatment, $F_{1, 14} = 2.378$, $P = 0.1453$, or time point $F_{2.798, 39.17} = 1.361$, $P = 0.269$; Day 4, no main effect of treatment, $F_{1, 14} = 2.707$, $P = 0.0221$, or time point $F_{2.798, 39.17} = 1.361$, $P = 0.3784$; Day 10, no main effect of treatment, $F_{1, 14} = 1.803$, $P = 0.2007$, or time point $F_{2.394, 33.51} = 0.854$, $P = 0.4501$. Vehicle $N = 7$, JZL184 $N = 9$). (**I**) Experimental timeline of acute systemic vehicle or PF-3845 pretreatment prior to the morphine dose response in the tail-flick test. (**J**) The ED_{50} value of systemic PF-3845 pretreated animals were comparable to that in animals that received vehicle. (Two-way ANOVA, main effect of dose, $F_{4, 80} = 44.34$, $P < 0.001$; Vehicle $N = 11$, PF-3845 $N = 7$). Error bars ± SEM.

Supplementary Fig. 4

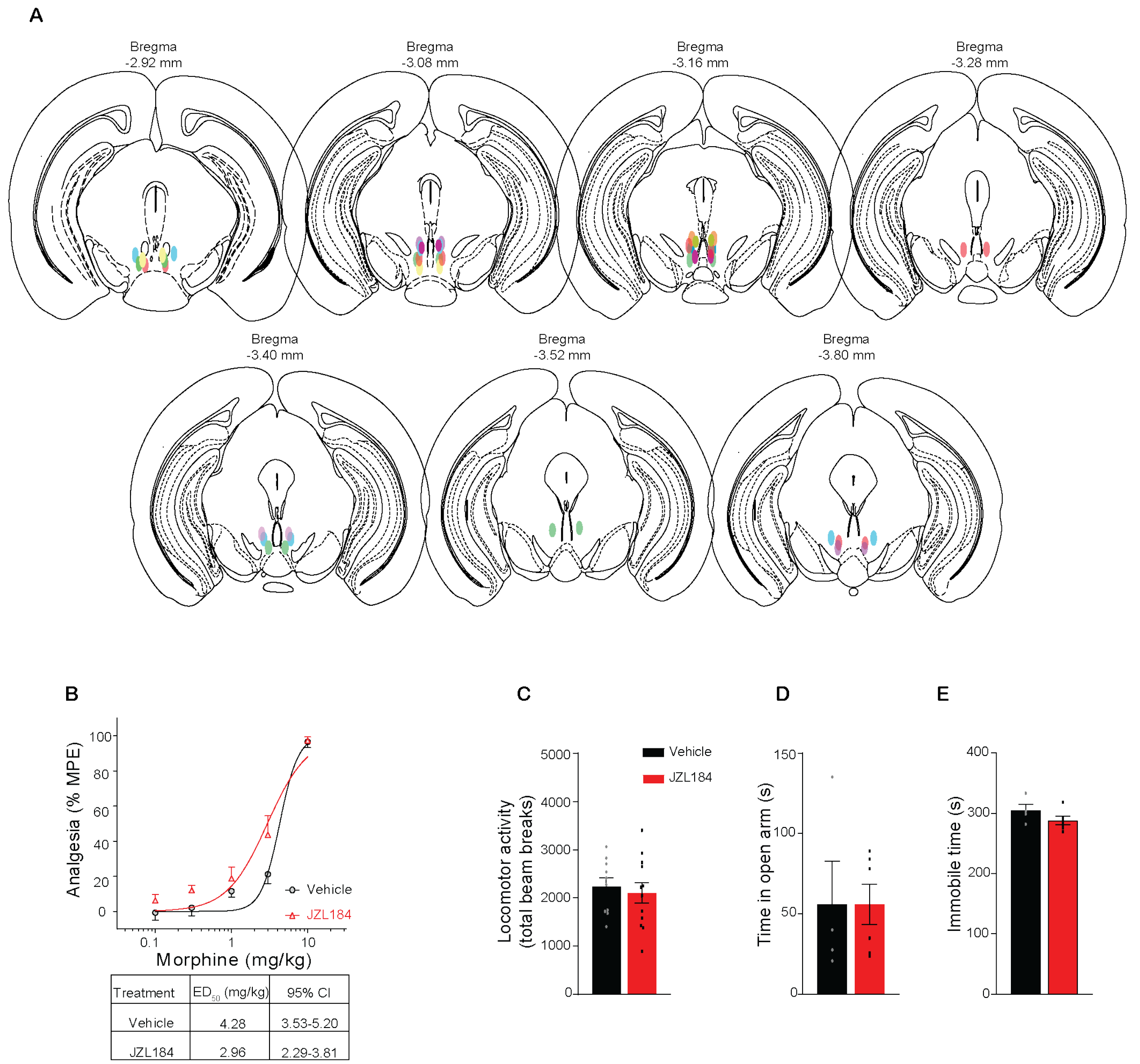

Treatment	ED_{50} (mg/kg)	95% CI
Vehicle	4.28	3.53-5.20
JZL184	2.96	2.29-3.81

Fig. S4. Brain placements and behavioral data for intra-VTA JZL184 treated mice. (**A**) Coronal sections of JZL184 guide cannula placement in the VTA. Each animal received bilateral implantation, and the placement of animal's bilateral cannula is represented by colored circles. Some of the circles represent multiple animals, as placement overlapped across animals. (**B**) Intra-VTA JZL184 does not inhibit morphine analgesia (Two-way ANOVA, main effect of dose, $F_{4,105} = 107.7$, $P < 0.001$, Vehicle $N = 11$, JZL184 $N = 12$). (**C**) Locomotor activity on morphine CPP test day is not significantly different between intra-VTA vehicle and JZL184 pretreated mice (Ttest, $t_{(20)} = 0.4685$, $P = 0.6445$, Vehicle $N = 10$, JZL184 $N = 12$). (**D, E**) Time in the open arm of the elevated plus maze (**D**; Ttest, $t_{(8)} = 0.000$, $P > 0.999$, Vehicle $N = 4$, JZL184 $N = 4$), and immobile time in

the tail suspension test (**E;** Ttest, $t_{(8)} = 1.357$, $P = 0.2119$, Vehicle $N = 4$, JZL184 $N = 4$) is not significantly different between intra-VTA vehicle and JZL184 pretreated mice.

Supplementary Fig. 5

A

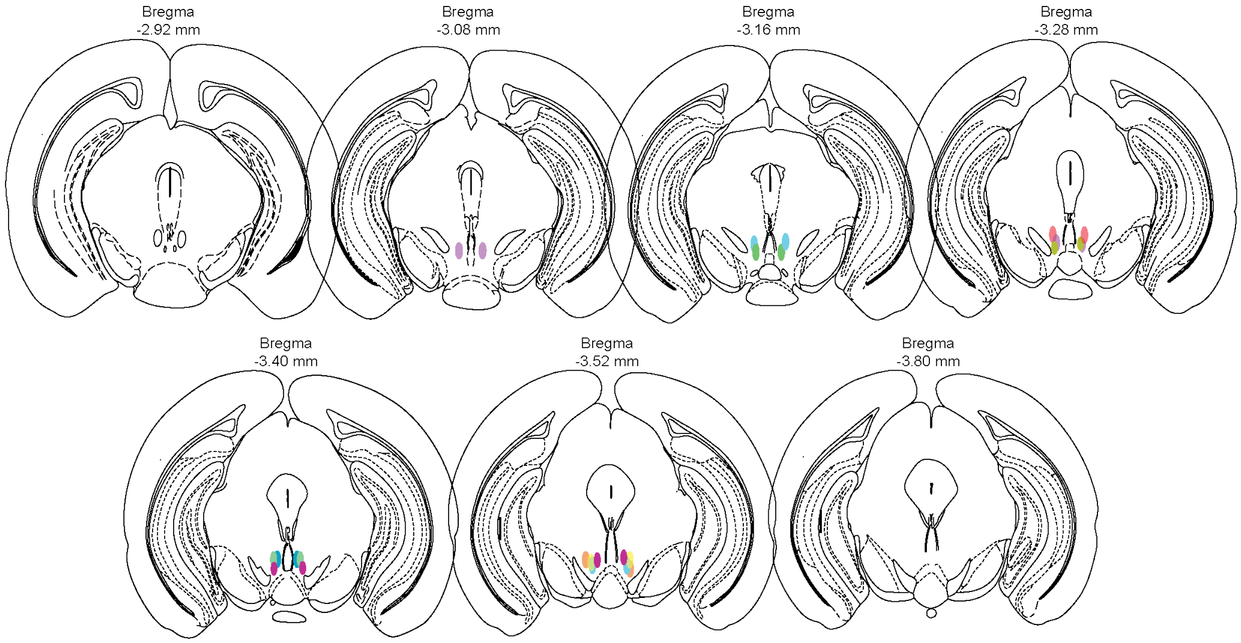

B

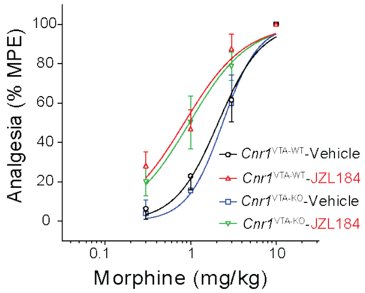

Treatment	ED$_{50}$ (mg/kg)	95% CI
Cnr1^{VTA-WT}-Vehicle	2.11	1.58-2.80
Cnr1^{VTA-WT}-JZL184	0.84	0.59-1.20
Cnr1^{VTA-KO}-Vehicle	2.38	1.74-3.27
Cnr1^{VTA-KO}-JZL184	0.97	0.65-1.44

Fig. S5. Brain placements and analgesia response in VTA CB1R knockout male mice (**A**) Coronal sections of AAV2-GFP-Cre expression in the VTA of *Cnr1* floxed mice. Animal's viral expression location is represented by different colored circles. Viral placements for some animals overlapped and thus some of the circles represent multiple animals. (**B**) Focal knockout of CB1Rs in the VTA does not inhibit morphine analgesia effects (Three-way ANOVA, no genotype main effect or interaction, main effect of treatment JZL184 vs Vehicle $F_{1,60} = 10.73$, $P < 0.0019$, and significant interaction of Treatment (JZL184 or Vehicle) x dose, $F_{3,180} = 3.601$, $P < 0.015$; (*Cnr1*

$^{VTA\text{-}WT}$ Vehicle *N* = 9, *Cnr1* $^{VTA\text{-}WT}$ JZL184 *N* = 9, *Cnr1* $^{VTA\text{-}KO}$ Vehicle *N* = 7, *Cnr1* $^{VTA\text{-}KO}$ JZL184 *N* = 11). Error bars ± SEM.

Supplementary Fig. 6

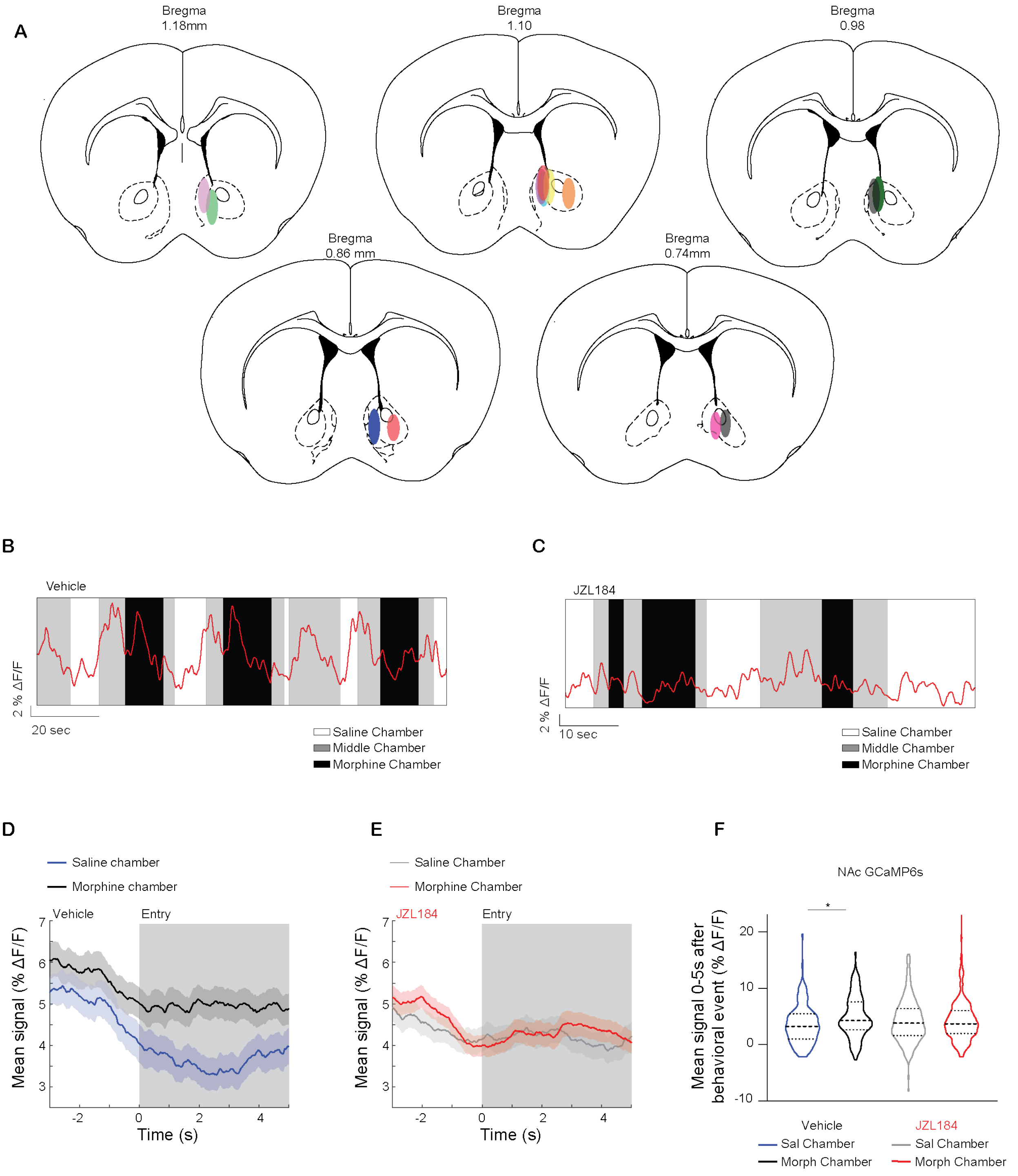

Fig. S6. Brain placements and behavioral NAc GCaMP fiber photometry recordings (A) Coronal sections of the fiber implant and GCaMP6s viral expression in the NAc. The placement of animal's unilateral fiber cannula and viral expression is represented by different colored circles. The placements of some animals overlapped and thus some circles include more than one animal. **(B-C)** Representative GCaMP6s traces collected during the CPP preference test day 6 in vehicle (**B**) or

JZL184 (**C**) pretreated animals. (**D**) Average trace of NAc activity in vehicle pretreated animals time-locked to entry into the morphine-paired chamber (black) or the saline-paired chamber (blue). Grey shading indicates window used for quantification in H. $N = 6$ mice, 113 morphine chamber entries, 135 saline chamber entries. (**E**) Average trace of NAc activity in JZL184 pretreated animals time-locked to entry into the morphine-paired chamber (red) or the saline-paired chamber (grey). Grey shading indicates window used for quantification in H. $N = 9$ mice, 198 morphine entries, 239 saline entries. (**F**) Mean NAc signal during entry (0-5s window) into the saline- or morphine-paired chamber in vehicle or JZL184 pretreated animals. In vehicle animals, there was a significant difference in signal during entry into morphine- compared to saline-paired chambers ($N = 6$ mice, 113 morphine chamber entries, 135 saline chamber entries; Linear mixed effect model – ANOVA marginal test, significant effect of group $F_{1,246} = 7.8$; ***$P = 0.006$). There was no significant difference in signal in the JZL184 pretreated animals ($N = 9$ mice, 198 morphine entries, 239 saline entries; Linear mixed effect model – ANOVA marginal test, no effect of group $F_{1,435} = 0.05$; $P = 0.83$). Error bars ± SEM (of trials).

Supplementary Fig. 7

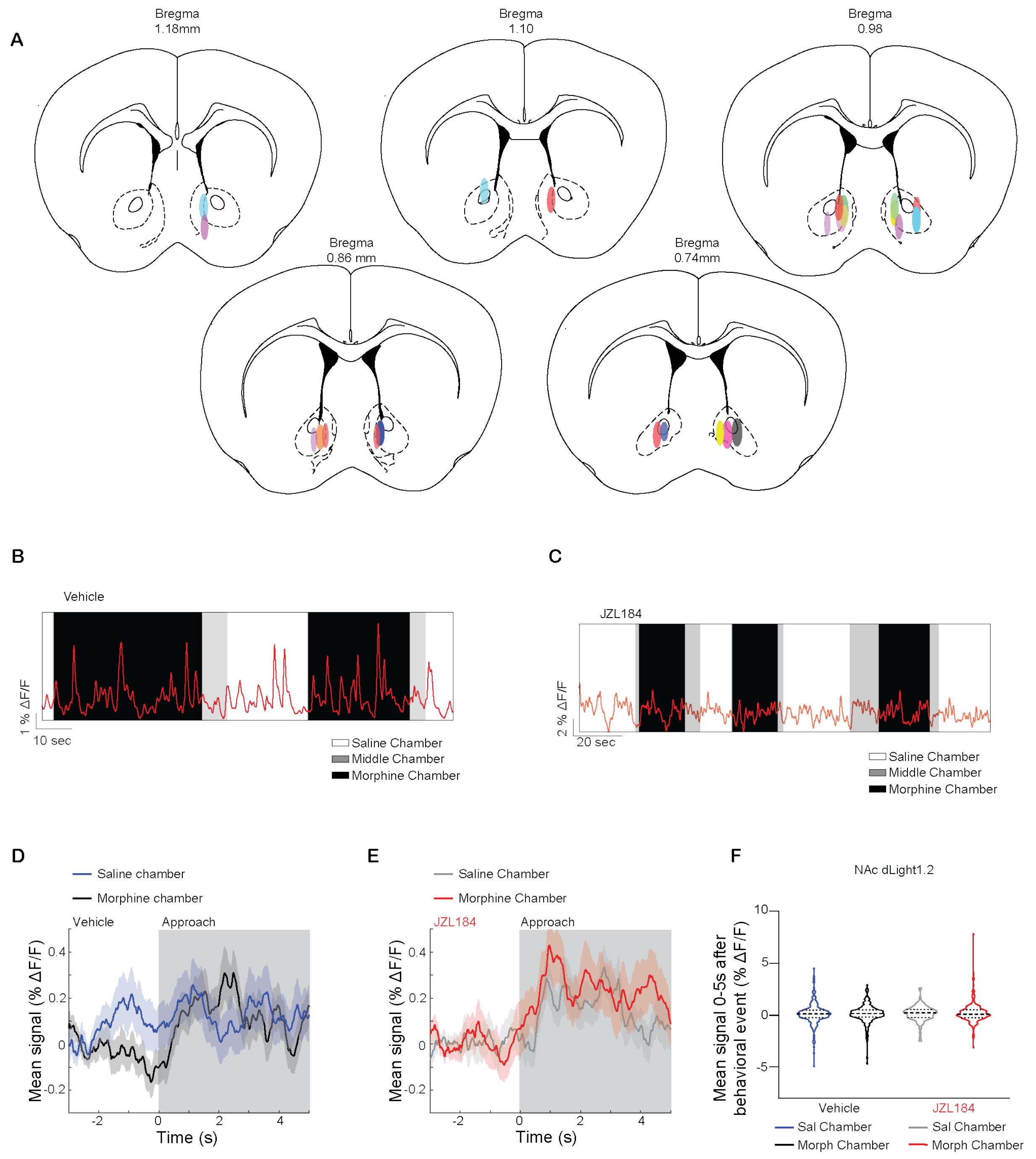

Fig. S7. Brain placements and behavioral NAc dLight1.2 fiber photometry recordings (A) Coronal sections of the fiber implant and dlight1.2 viral expression in the NAc. The placement of animal's unilateral fiber cannula and viral expression is represented by different colored circles. The placements of some animals overlapped and thus some circles include more than one animal. (**B-C**) Representative dlight1.2 traces collected during the CPP preference test day 6 in vehicle (**B**) or JZL184 (**C**) pretreated animals. (**D**) Average trace of DA signal in vehicle pretreated animals time-locked to approach of the morphine-paired chamber (black) or the saline-paired chamber (blue).

Grey shading indicates window used for quantification in F. *N* = 16 mice, 236 morphine chamber approaches, 232 saline chamber approaches. (**E**) Average trace of DA signal in JZL184 pretreated animals time-locked to approach of the morphine-paired chamber (red) or the saline-paired chamber (grey). Grey shading indicates window used for quantification in F. *N* = 12 mice, 184 morphine chamber approaches, 176 saline chamber approaches. (**F**) Mean DA signal during approach (0-5s window) of saline- or morphine-paired chambers in vehicle or JZL184 pretreated animals. In vehicle animals, there was no significant difference in signal during approach of morphine- compared to saline-paired chambers (*N* = 16 mice, 236 morphine chamber approaches, 232 saline chamber approaches.; Linear mixed effect model – ANOVA marginal test, no effect of group $F_{1,466} = 0.03$; $P = 0.87$). There was also no significant difference in signal in the JZL184 pretreated animals (*N* = 12 mice, 184 morphine chamber approaches, 176 saline chamber approaches; Linear mixed effect model – ANOVA marginal test, no effect of group $F_{1,358} = 0.79$; $P = 0.37$). Error bars ± SEM (of trials).
